# Experimental Oral Herpes Simplex Virus-1 (HSV-1) Co-infection in Simian Immunodeficiency Virus (SIV)-Infected Rhesus Macaques

**DOI:** 10.3389/fmicb.2017.02342

**Published:** 2017-12-05

**Authors:** Meropi Aravantinou, Olga Mizenina, Giulia Calenda, Jessica Kenney, Ines Frank, Jeffrey D. Lifson, Moriah Szpara, Lichen Jing, David M. Koelle, Natalia Teleshova, Brooke Grasperge, James Blanchard, Agegnehu Gettie, Elena Martinelli, Nina Derby

**Affiliations:** ^1^Center for Biomedical Research, Population Council, New York, NY, United States; ^2^AIDS and Cancer Virus Program, Leidos Biomedical Research, Inc., Frederick National Laboratory for Cancer Research, Frederick, MD, United States; ^3^Departments of Biochemistry and Molecular Biology, Pennsylvania State University, University Park, PA, United States; ^4^Department of Medicine, University of Washington, Seattle, WA, United States; ^5^Vaccine and Infectious Disease Division, Fred Hutchinson Cancer Research Center, Seattle, WA, United States; ^6^Department of Laboratory Medicine, University of Washington, Seattle, WA, United States; ^7^Department of Global Health, University of Washington, Seattle, WA, United States; ^8^Benaroya Research Institute, Seattle, WA, United States; ^9^Tulane National Primate Research Center, Tulane University Health Sciences Center, Covington, LA, United States; ^10^Aaron Diamond AIDS Research Center, Rockefeller University, New York, NY, United States

**Keywords:** HSV-1, SIV, non-human primate model, oral infection, immune response

## Abstract

Herpes simplex virus 1 and 2 (HSV-1/2) similarly initiate infection in mucosal epithelia and establish lifelong neuronal latency. Anogenital HSV-2 infection augments the risk for sexual human immunodeficiency virus (HIV) transmission and is associated with higher HIV viral loads. However, whether oral HSV-1 infection contributes to oral HIV susceptibility, viremia, or oral complications of HIV infection is unknown. Appropriate non-human primate (NHP) models would facilitate this investigation, yet there are no published studies of HSV-1/SIV co-infection in NHPs. Thus, we performed a pilot study for an oral HSV-1 infection model in SIV-infected rhesus macaques to describe the feasibility of the modeling and resultant immunological changes. Three SIV-infected, clinically healthy macaques became HSV-1-infected by inoculation with 4 × 10^8^ pfu HSV-1 McKrae on buccal, tongue, gingiva, and tonsils after gentle abrasion. HSV-1 DNA was shed in oral swabs for up to 21 days, and shedding recurred in association with intra-oral lesions after periods of no shedding during 56 days of follow up. HSV-1 DNA was detected in explant cultures of trigeminal ganglia collected at euthanasia on day 56. In the macaque with lowest baseline SIV viremia, SIV plasma RNA increased following HSV-1 infection. One macaque exhibited an acute pro-inflammatory response, and all three animals experienced T cell activation and mobilization in blood. However, T cell and antibody responses to HSV-1 were low and atypical. Through rigorous assessesments, this study finds that the virulent HSV-1 strain McKrae resulted in a low level HSV-1 infection that elicited modest immune responses and transiently modulated SIV infection.

## Introduction

Herpes simplex virus (HSV) types 1 and 2 (HSV-1, HSV-2) are closely related alphaherpesviruses (Roizman et al., 2007). HSV-1 typically initiates a productive lytic infection in the oral mucosal epithelium and then establishes latency in the trigeminal ganglia after infecting sensory nerve termini adjacent to the site of mucosal virus replication. Occasional reactivation can result in recurrent lytic infection in the oral skin and mucosa with virus shedding into the saliva and in some instances, oral lesions. Infection with HSV-1 is ubiquitous in humans; more than 50% of adults in the US are HSV-1 seropositive, and close to 100% are infected in some parts of the developing world (Looker et al., 2015). Globally, most HSV-1 infections are oral although genital HSV-1 infection is on the rise, especially in the US and Europe (Looker et al., 2015). Oral HSV-1 infection is usually innocuous aside from being painful during recurrences, but there are a few potentially serious complications. HSV-1 infection can lead to corneal HSV keratitis, representing the most common cause of infection-related blindness worldwide, and rarely encephalitis in adults and children.

The immune response to HSV infection is multifaceted and capable of readily, if incompletely, controlling mucosal infection (Shin and Iwasaki, 2013). Acute pro-inflammatory and antiviral responses and chemokine gradients restrict the infection and recruit immune cells. Upon T cell priming, HSV-specific T cells migrate into the sites of recurrent mucocutaneous HSV infection where they are critical for controlling virus replication and where they persist at the dermo-epidermal junction (Zhu et al., 2007; Iijima et al., 2008). In the context of genital HSV-2 infection, some of the cells persisting in mucosal tissue during the immune response and after lesion clearance have a phenotype consistent with being permissive to human immunodeficiency virus (HIV; e.g., activated CD4^+^CCR5^+^ T cells and dendritic cells expressing HIV receptors) (Zhu et al., 2009). This is an important mechanism by which genital HSV-2 infection predisposes to HIV acquisition (Zhu et al., 2009; Martinelli et al., 2011; Goode et al., 2014; Shannon et al., 2014).

The study of HSV infection and immunity has been aided by the willingness of HSV-infected volunteers to be biopsied during episodes of reactivation and to have their lesions followed through the healing process (Zhu et al., 2007, 2009; Kim et al., 2015). However, primary infection is more difficult to document in humans, so most of what is known about primary infection and all that is known about priming of adaptive immunity comes from studies in mice and other small mammals (Kollias et al., 2015). Mice are usually experimentally infected with HSV-1 through the ocular route after corneal scarification although other models such as atraumatic oral, nasal, and esophageal infection; skin infection following stripping of the stratum corneum; and injection of gingival submucosa have been utlilized (Gesser and Koo, 1996; Monma et al., 1996; Puttur et al., 2010; Shivkumar et al., 2013; Kollias et al., 2015). However, HSV does not readily reactivate in mice, and the mouse immune system differs substantially from that of humans. HSV-1 transmission, pathogenesis, and vaccine studies would benefit from a non-human primate (NHP) model. Mechanistic studies on the role of oral HSV-1 infection in oral HIV transmission and pathogenesis will require a NHP model of co-infection with HSV-1 and pathogenic simian immunodeficiency virus (SIV). SIV infection of old world NHPs, such as rhesus and pigtail macaques, has proven invaluable for dissecting the mechanisms underlying HIV susceptibility and pathogenesis.

There are only a few published models of HSV-2 infection in rhesus macaques. These are the Population Council vaginal and rectal HSV-2 infection models developed in collaboration with the Blanchard group at Tulane National Primate Research Center (TNPRC) (Crostarosa et al., 2009; Martinelli et al., 2011; Goode et al., 2014; Hsu et al., 2014; Kenney et al., 2014; Kizima et al., 2014; Guerra-Pérez et al., 2016; Derby et al., 2017) and a vaginal acute HSV-2 infection model from the Veazey group at TNPRC (Awasthi et al., 2017; Stanfield et al., 2017). Macaque models of SHIV and SIVmac239 co-infection with HSV-2 mimic the HSV-2-driven increase in HIV acquisition risk (Martinelli et al., 2011; Goode et al., 2014; Guerra-Pérez et al., 2016).

NHP models of HSV-1 infection are largely in new world species: squirrel monkeys (by corneal scarification and infection with strains McKrae or Rodanus) (Kaufman, 1963; Varnell et al., 1987, 1995; Rootman et al., 1990); and owl monkeys and marmosets (by intracerebral injection with strain F) (Katzin et al., 1967; Hunter et al., 1999; Deisboeck et al., 2003). Tree shrews, which are prosimians, are susceptible ocularly to HSV-1 17+ after scarification and atraumatically with McKrae (Li et al., 2016). However, these models result in acute dendritic keratitis or encephalitis following HSV-1 infection, and new world NHPs are not susceptible to SIV. Thus, these models are inadequate for studying HSV-1 immunology as it relates to humans or HSV-1/SIV co-infection. Only one recent report described oral HSV-1 infection in rhesus macaques after lip scarification (Fan et al., 2017), and we recently described genital HSV-1 infection in SHIV-infected macaques (Aravantinou et al., 2017).

We sought to perform a pilot study to rigorously assess the feasibility of establishing a model of oral HSV-1 infection in SIV-infected rhesus macaques and to describe the resultant immunological changes. Such a model would facilitate studying HSV-1 biology, testing prevention strategies, and investigating if oral HSV-1 infection participates in HIV pathogenesis systemically and in the oral mucosa. As a proof of concept that indeed this co-infection was possible and to begin to evaluate the immunological consequences of HSV-1 oral infection in SIV-infected macaques, we infected three SIV-infected macaques with HSV-1 orally with only mild abrasion and observed primary infection along with recurrence during 56 days of follow up. Mucosal innate and systemic T cell activation were observed, but HSV-specific T cell and antibody responses did not develop efficiently. However, oral HSV-1 infection did modulate SIV viremia orally and systemically, suggesting interplay between the infections.

## Methods

### Animals

Five adult female Indian rhesus macaques (*Macaca mulatta*; mean age: 10 years, range: 6–15 years; mean weight: 7.2 kg, range: 5.4–10.0 kg) that tested negative by serology for simian retrovirus (SRV), Herpes virus B, and simian T cell leukemia virus type 1 (STLV-1) were selected for these studies. All macaques were chronically infected with SIVmac251, having been inoculated intrarectally with 10 half-maximal animal infectious doses (AID_50_) as part of another study more than 1 year prior to initiating this study (Table 1) (Andrews et al., 2015).

**Table 1:** Characteristics of HSV-1 and SIV co-infection in rhesus macaques.

**Macaque ID**	**Baseline parameters**			**HSV-1 challenge phase parameters**							
	**SIV^a^ plasma VL (RNA copies/ml)**	**SIV oral VL (DNA copies/10^6^ cells)^b^**	**CD4 Count (d-14, d0)^d^**	**HSV-1 Strain, Dose (pfu)**	**Challenge route**	**Oral shedding**	**TG^f^ Infection**	**SIV plasma VL (d14, d56)**	**SIV oral VL (DNA copies/10^6^ cells**	**CD4 Count post- HSV-1**	**Clinical profile^g^**
EL75	n/a	n/a	n/a^e^, 404	F, 4 × 10^8^	Intra-oral, atraumatic	d21	ND	n/a	n/a	d21: 251	Normal
FB90	n/a	n/a	n/a, 862	F, 1 × 10^9^	Intra-oral, atraumatic	None	ND	n/a	n/a	d21: 605	Normal
IB13	860	Bc: ND^c^ Tn: 1.6 × 10^3^	348, 594	McKrae, 4 × 10^8^	Intra-oral, cytobrush	Extended	Yes	1.5 × 104, 1.6 × 10^3^	Bc: ND Tn: ND	d28: 647 d56: 752	Periodontal hemorrhage (d1), enlarged LNs (d3, 7, 14), poor appetite (d7-14), bloody nasal discharge (d28), high neutrophils WBCs (d28, 42), lesion right cheek buccal (d28, 35, 56)
DF98	9.3 × 10^4^	Bc: 71 Tn: 2.6x10^4^	305, 383	McKrae, 4 × 10^8^	Intra-oral, cytobrush	Extended	Possible	9.7 × 104, 1.1 × 10^5^	Bc: ND Tn: 1.3 × 10^4^	d28: 300 d56: 251	Normal
GJ18	3.2 × 10^5^	Bc: 545 Tn: 2.4 × 10^4^	103, 85	McKrae, 4 × 10^8^	Intra-oral, cytobrush	Extended	ND	7.3 × 105, 5.2 × 10^5^	Bc: 1.8 × 103 Tn: 2.1 × 10^4^	d28: 108 d56: 111	Mild periodontal hemorrhage (BL), gingival ulcers (d2), left cheek lesion (d3, 7), left labial mucosa lesion (d14)

a *All animals were infected >1 year prior with 10 half-maximal animal infectious doses (AID_50_) SIVmac251 as part of a separate study (Andrews et al. 2015). SIV viral loads were not measured for EL75 and FB90 before or during the study and so were not available (n/a)*.

b *Bc, Buccal; Tn, Palatine tonsil*.

c *ND, not detected. SIV DNA was below the lower limit of detection of the assay*.

d *d-14 indicates 2 weeks before HSV-1 challenge. d0 indicates the day of HSV-1 challenge*.

e *n/a, For EL75 and FB90, only d0 data were available*.

f *TG, trigeminal ganglia*.

g *LN, lymph node; WBC, white blood cell; BL, baseline*.

### Ethics statement

Animal care complied with the regulations stated in the Animal Welfare Act and the Guide for the Care and Use of Laboratory Animals, at TNPRC (Covington, LA) (1992, Ackerman et al., 2011). All macaque studies were approved by the Institutional Animal Care and Use committee (IACUC) of TNPRC for macaques (OLAW Assurance #A4499-01) and complied with TNPRC animal care procedures. TNPRC receives full accreditation by the Association for Accreditation of Laboratory Animal Care (AAALAC #000594). Animals were socially housed indoors in climate-controlled conditions and were monitored twice daily by a team of veterinarians and technicians to ensure their welfare. Any abnormalities, including changes in appetite, stool, and behavior, were recorded and reported to a veterinarian. They were fed commercially prepared monkey chow twice daily. Supplemental foods were provided in the form of fruit, vegetables, and foraging treats as part of the TNPRC environmental enrichment program. Water was available continuously through an automated watering system.

Veterinarians at the TNPRC Division of Veterinary Medicine have established procedures to minimize pain and distress through several means in accordance with the recommendations of the Weatherall Report. Prior to all procedures, including blood collection, macaques were anesthetized with ketamine-HCl (10 mg/kg) or tiletamine/zolazepam (6 mg/kg). Preemptive and post-procedural analgesia (buprenorphine 0.01 mg/kg) was administered for procedures that could cause more than momentary pain or distress in humans undergoing the same procedures. All macaques were euthanized at the conclusion of the study using methods consistent with recommendations of the American Veterinary Medical Association (AVMA) Guidelines for the Euthanasia of Animals and per the IACUC protocol. For euthanasia, animals were anesthetized with tiletamine/zolazepam (8 mg/kg) and given buprenorphine (0.01 mg/kg) followed by an overdose of pentobarbital sodium. Death was confirmed by auscultation of the heart and pupillary dilation.

### Viruses

HSV-1 strains F and McKrae were used for these studies. HSV-1 F was obtained from ATCC. HSV-1 McKrae was a fully sequenced plaque purified stock from the Szpara laboratory. The stock was originally from Dr. Lynda Morrison (Macdonald et al., 2012). To expand virus stocks, Vero cells were inoculated with 0.6 MOI HSV-1 in Dulbecco's modified Eagle medium (DMEM, Cellgro Mediatech, Manassas, VA) for 3 hours and cultured overnight in DMEM with 2% fetal bovine serum (FBS, Gibco Life Technologies, Grand Island, NY) and 100 IU penicillin/100 μg/ml streptomycin (VWR, Radnor, PA). Monolayers were scraped, and the cells washed, twice frozen and thawed in a dry ice/ethanol bath, and sonicated before supernatant containing virus was cleared by centrifugation, aliquotted, and stored at −80°C. Titer was determined by plaque assay as follows. Virus dilutions were incubated with Vero cells for 1 h and overlayed with methylcellulose (Sigma-Aldrich, St. Louis, MO) for 48 h. Cells were fixed in 10% formalin (Sigma), stained with 1% crystal violet (Sigma), washed, dried, and the plaques counted. Growth of HSV-1 F yielded a stock 4 × 10^9^ pfu/ml. Growth of HSV-1 McKrae yielded a stock 8 × 10^9^ pfu/ml.

### *Ex vivo* HSV-1 challenge of macaque oral tissues

3 × 3 × 3 mm buccal and tonsillar explants were exposed to HSV-1 F (10^3^-10^6^ pfu/explant) in DMEM containing 10% FBS, 100 IU penicillin/100 μg/ml streptomycin, and 100 μM minimal essential media (MEM) non-essential amino acids (Irvine Scientific, Santa Ana, CA) for 18 h. An acyclovir (ACV, EDM Millipore, Darmstadt, GER) control condition was included for each tested viral dose (0.02 mg/well). After incubation, tissues were washed six times in PBS (Thermo Fisher, Waltham, MA) and cultured for 14 days. Tissue culture supernatants were collected on days 1, 3, 7, 11, and 14 for monitoring infection by plaque assay, as described above.

### Oral HSV-1 challenge and specimen collection

Atraumatic oral inoculation was attempted in two macaques chronically infected with SIVmac251 using HSV-1 strain F (Table 1). One animal (EL75) received 4 × 10^8^ pfu, and the other (FB90) received 1 × 10^9^ pfu. The oral cavity was held open, epithelia were dried, and virus diluted in saline to a total volume of 1 ml was dribbled over all the epithelial surfaces inside the mouth including the buccal, gingival, and sublingual mucosae and the epithelia covering the palatine, pharyngeal, and lingual tonsils, targeting the crypt epithelia in a manner similar to that used for tonsillar SIV challenge (Stahl-Hennig et al., 1999; Jasny et al., 2012). Animals were maintained reclining for 5 min to encourage virus binding. Blood was drawn into ethylenediaminetetraacetic acid (EDTA)-coated tubes just prior to challenge (baseline) and then on days 3, 7, 14, and 21. Oral swabs were also collected at these times using a Merocel eye spear (Medtronic Xomed, Jacksonville, FL). The swab was pre-moistened in 1 ml PBS/1% FBS, inserted in the cheek pouch for 5 min to absorb liquid, and rotated to brush the epithelia before being returned to the tube containing PBS/FBS. On day 21 post-infection, the macaques were euthanized, and the following tissues were collected at the necropsy and snap frozen: mucosal (buccal, gingiva, sublingual), lymphoid (pharyngeal, palatine, and lingual tonsils; submandibular, retropharyngeal, and cervical lymph nodes), and neural (trigeminal ganglia).

Three other macaques chronically infected with SIVmac251 (IB13, DF98, and GJ18) were inoculated with 4 × 10^8^ pfu HSV-1 McKrae (similarly prepared in 1 ml saline) after gentle cytobrushing of the oral epithelia (Table 1). Cytobrushing entailed applying a Medscand cytobrush plus (Medscand Medical, Trumbull, CT) to the buccal, gingival, and sublingual mucosae and the epithelia covering the palatine, pharyngeal, and lingual tonsils and sweeping over tissues for five rotations. Virus was then applied to the mucosal and tonsillar epithelia inside the mouth as in the first trial with HSV-1 F. EDTA blood was drawn at baseline and then on days 1, 2, 3, 7, 14, 21, 28, 35, 42, 49, and 56. Oral swabs were collected at these times as above. Blood was drawn into tubes coated with clot activator on day 56 to obtain serum. Buccal mucosa (3 × 3 mm) and palatine tonsil (1.5 × 1.5 mm) were biopsied 2 weeks prior to challenge and more tissue (3 × 3 mm from both sites) was collected at the time of euthanasia, day 56 post-infection. Lesions observed during the study were also biopsied. These tissues were all snap frozen. At euthanasia, a portion of trigeminal ganglia was snap frozen, and another portion was placed in L-15 media (GE Healthcare, Logan, UT). Spleens were harvested and also placed in L-15.

All biological samples were shipped to the Population Council laboratories in New York overnight and were processed immediately upon arrival. Blood, swabs, and tissues in L-15 were shipped cold, and snap frozen tissues were shipped on dry ice. Plasmas were isolated and stored at −80°C as previously described (Aravantinou et al., 2016). PBMCs, isolated as previously described (Aravantinou et al., 2016), were used immediately for flow cytometry or were cryopreserved in freezing medium consisting of Roswell Park Memorial Institute medium (RPMI) 1640 (Thermo Fisher) with 50% FBS and 10% DMSO (Sigma). Splenocytes were isolated by manual dissociation and passage through a 40 μm cell strainer and then cryopreserved in freezing medium. Oral swabs were pipetted to mix, and a portion of the cell/fluid mixture was aliquotted as “unclarified” swab. The remainder of the swab was centrifuged and the cell-free liquid (“clarified” swab) and pellet were stored separately. All swab aliquots were kept at −80°C.

### HSV shedding

DNA was extracted from unclarified oral swabs, snap frozen tissues, and explanted trigeminal ganglia cultures using the DNeasy mini kit (Qiagen, Hilden, GER, used by manufacturer's instructions). The DNA was quantified on a Nanodrop 1000 spectrophotometer (Thermo Scientific, Wilmington, DE) and subjected to nested PCR (nPCR) in US6 (gD gene) of HSV-1. Maximal amount of DNA (mean ± standard deviation: 199 ± 121 ng) was included in each reaction. The primers were as follows: External Forward 5′ ATC ACG GTA GCC CGG CCG TGT GAC A 3′; External Reverse 5′ CAT ACC GGA ACG CAC CAC ACA A 3′; Internal Forward 5′ CCA TAC CGA CCA CAC CGA CGA 3′; Internal Reverse 5′ GGT AGT TGG TCG TTC GCG CTG AA (Aurelius et al., 1991). The positive control was HSV-1 MacIntyre Quantitated Viral Load Control (Advanced Biotechnologies, Eldersburg, MD). Nested PCR products from some reactions were sequenced using the forward nesting primer to confirm amplicon identity (Genewiz, South Plainfield, NJ). A heat map was generated by assigning grades of color intensity to indicate the frequency of positive amplicons from replicate nPCR reactions, both as a function of the total number of six PCR replicates and the amount of total DNA in each reaction, as this varied.

Quantitative PCR (qPCR) in UL27 (gB gene) was performed according to the published protocol (Jerome et al., 2002) optimized by Dr. Szpara for background reduction with a double quenched probe 5′-/56-FAM/CCACGAGAT/ZEN/CAAGGACAGCGGCC/3′IBFQ/-3′ using DNA extracted (by Qiagen DNeasy kit) from oral swab pellets. DNA extracted from the HSV-1 MacIntyre Quantitated Viral Load Control was diluted into a standard curve for the qPCR. The reactions were prepared in Taqman Universal PCR Master Mix (Roche, Indianapolis, IN) and run on a ViiA 7 (Thermo Fisher). The lower limit of quantification of the assay was below 4.4 genomes/μl. Samples and standards were run in duplicate or triplicate.

### Trigeminal ganglia explants

Trigeminal ganglia were explanted in MEM (Thermo Fisher) as previously described (Sawtell and Thompson, 2004). The cultures were harvested after 24 h and freeze-thawed twice before the tissues were disrupted mechanically with a mortar and pestle and sonicated. The mixtures were centrifuged to give a clarified supernatant from which DNA was extracted and subjected to nPCR in US6. The PCR product was sequenced.

### SIV viral load

For plasma, RNA copies of SIV were determined by reverse transcriptase qPCR (RT-qPCR) as previously described (Cline et al., 2005; Aravantinou et al., 2016). The lower limit of quantification of the assay was 30 RNA copies/ml. Primers and probe were SGAG21 (forward), 5′-GTC TGC GTC ATP TGG TGC ATT C-3′; SGAG22 (reverse), 5′-CAC TAG KTG TCT CTG CAC TAT PTG TTT TG-3′; and pSGAG23 (probe, 100 nM), 5′-(FAM) CTT CPT CAG TKT GTT TAC TTT CTC TTC TGC G-(BHQTM1)-3′.

For PBMCs and biopsies of buccal mucosa and palatine tonsil, DNA copies of SIV were measured. Total DNA was isolated from PBMC dry pellets (1–5 × 10^6^ cells) and tissue biopsies using the DNeasy mini kit and quantified on the Nanodrop. No more than 250 ng of DNA was used in Taqman qPCR and analyzed by the standard curve method with albumin as the cell control, as previously described (Guerra-Pérez et al., 2016; Aravantinou et al., 2017). Primers were SIVgag667 FW: 5′–GGT TGC ACC CCC TAT GAC AT; SIVgag731REV: 5′–TGC ATA GCC GCT TGA TGG T; SIVgag 688 PROBE: 5′–/56 FAM/ATT CAG ATG TTA AAT TGT GTG; RhAlb FW: 5′–ATT TTC AGC TTC GCG TCT TTT; RhAlb RV: 5′–TTC TCG CTT ACT GGC GTT TTC T; RhAlb PROBE: 5′–/56-FAM/CCT GTT CTT TAG CTG TCC GTG. For PBMCs, no day 56 sample was available.

### Luminex

The Novex® Monkey Cytokine Magnetic 29-Plex Panel kit (Life Technologies, Carlsbad, CA) was used on a MAGPIX® system (Luminex XMAP Technology, Luminex, Austin, TX) with Luminex xPOPNENT software to measure cytokines, chemokines, and growth factors. Clarified plasma and oral swab supernatants were thawed from −80°C, spun, and aliquotted neat in the assay with 1:3 final dilution. The assay was performed according to the manufacturer's instructions. The soluble factors measured were as follows: interleukin 1β (IL-1β), IL-1 receptor antagonist (IL-1RA), IL-2, IL-4, IL-5, IL-6, IL-10, IL-12, IL-15, IL-17, granulocyte-colony stimulating factor (G-CSF), granulocyte macrophage colony stimulating factor (GM-CSF), interferon-γ (IFN-γ), tumor necrosis factor-α (TNF-α), chemokine (C-C motif) ligand 2 (CCL2), CCL3, CCL4, CCL5, CCL11, CCL22, chemokine (C-X-C motif) ligand 8 (CXCL8), CXCL9, CXCL10, CXCL11, macrophage migration inhibitory factor (MIF), fibroblast growth factor-basic (FGF), epidermal growth factor (EGF), vascular endothelial growth factor (VEGF), and hepatocyte growth factor (HGF). Values above the lower limit of detection (lowest standard concentration) for each analyte were plotted. Values below the lowest standard concentration were plotted as the lower limit of detection. No values fell above the range of the standard curve.

### Flow cytometry

#### For T cell phenotyping

Monoclonal antibodies (mAbs, listed as follows) were all mouse anti-human from BD Biosciences (Franklin Lakes, NJ) unless noted. All clones were known to cross-react with rhesus macaque or were confirmed cross-reactive and titrated before use: anti-CD3 AlexaFluor700 (clone SP34-2), anti-CD4 V450 (clone L200), anti-CD8 BUV395 (clone RPA-T8), anti-CD69 APC-H7 (clone FN50), anti-CCR7 BV605 (clone G043H7, Biolegend, San Diego, CA), anti-CD95 FITC (clone DX2), anti-α_4_β_7_ APC (clone A4B7, obtained through the NHP Reagent Resource), anti-CCR5 PE-Cy7 (clone 3A9, obtained through the NIH AIDS Reagent Program, Division of AIDS, NIAID, NIH, conjugated in-house using Lightening Link conjugation kit from Innova Biosciences, Babraham, UK), and anti-CCR6 PE (clone 11A9). Suspensions of PBMCs were stained with the LIVE/DEAD Fixable Aqua viability dye (Aqua; Molecular Probes, Life Technologies) according to the manufacturer's instructions. Surface staining was performed for 5 min at room temperature followed by 15 min at 4°C after which cells were washed, fixed in 2% paraformaldehyde, and washed again before acquisition on an LSRII (BD) using FACSDiva software (BD). The gating strategy for CD4^+^/CD8^+^ T cells was small cells (FSC-A/SSC-A) → singlets → live cells → CD4^+^ T cells/CD8^+^ T cells (CD3^+^CD4^+^/CD3^+^CD8^+^). On average, 60,000 CD3^+^ cells were acquired per sample. The data were analyzed with FlowJo software (TreeStar, Ashland OR).

#### For HSV-1-specific T cells

HSV-1-specific T cells in PBMCs and spleen were measured using the protocol our colleagues and we have used previously to measure SIV-specific T cells in rhesus macaque samples (Vagenas et al., 2010; Jasny et al., 2012; Hsu et al., 2014). PBMCs and splenocytes from day 56 were thawed, washed, and re-suspended at 7.5 × 10^6^ cells/ml in R10 media (RPMI 1640 with 100 IU penicillin/100 μg/ml streptomycin and 10% FBS) and incubated with antigens and co-stimulatory mAbs anti-CD28 (clone CD28.2) and anti-CD49d (clone 9F10, both 10 μg/ml, from BD). Co-stimulatory mAbs were first were cross-linked to the wells of a 96-well tissue culture plate via goat-anti-mouse F(ab)_2_ (2.5 μg/ml, from KPL, Gaithersburg, MD) (Gauduin et al., 2004; Vagenas et al., 2010). HSV antigens were UV-inactivated HSV-1 lysate and pools of overlapping 15-mer peptides spanning the immunodominant ORFs UL19 (major capsid protein, HSV-2 sequence cross-reactive with HSV-1, 170 peptides), UL39 (ribonucleoside-diphosphate reductase large subunit, HSV-1 sequence, 141 peptides), and US6 (gD glycoprotein, HSV-1 sequence, 128 peptides). Lysates were diluted 1:100 in the well, and peptides were at a final concentration of 1 μg/ml each. The negative control for HSV-1 lysate was mock lysate and for peptides was DMSO. The positive control was phorbol 12-myristate 13-acetate (Sigma, 20 ng/ml)/ionomycin (Sigma, 0.5 μg/ml) (PMA/iono). Following a 1 h incubation of antigens and co-stimulatory mAbs with cells, Brefeldin A (Sigma, 10 μg/ml) and monensin (Golgistop, BD, 0.5 μl stock/well) were added for 5 h, and the cells were washed and surface stained with Fixable viability stain 575V (BD) according to the manufacturer's instructions and then a panel of mouse anti-human mAbs prepared in Brilliant Buffer (mAbs and buffer from BD): anti-CD3 APC-Cy7 (clone SP34.2), anti-CD4 BUV-395 (clone L200), CD8-BUV496 (clone RPA-T8), anti-CD40L PE (clone TRAP1), and anti-CD69 PerCP-Cy5.5 (clone FN50) for 15 min dark 4°C. After washing, the cells were fixed in 1% paraformaldehyde (Electron Microscopy Sciences, Hatfield, PA) for 10 min and permeabilized in 1x Perm/Wash buffer (BD) for 20 min before intracellular staining was performed with the following panel of mAbs (from BD, all mouse anti-human unless noted): anti-IFN-γ PE-Cy7 (clone 4S.B3), anti-IL-2 APC-R700 (clone MQ1-17H12, rat), anti-TNF-α PE-CF594 (clone MAb11), and anti-IL-17A BV421 (clone N49-653) for 30 min dark at room temperature. After washing, the cells were analyzed immediately on the LSRII (BD). All mAbs for surface and intracellular labeling were known cross-reactive with macaque antigens or confirmed and titrated before use. The gating strategy and analysis to identify CD4^+^/CD8^+^ T cells was as described for phenotyping above. Gates around cytokine-expressing cells were set based on the populations observed with PMA/iono stimulation in the same macaques and the populations observed when cells from SIV-infected macaques in other studies were stimulated with a pool of peptides spanning SIVmac239 gag and stained according to the same protocol used herein (data not shown).

### ELISA for HSV-1-specific antibodies

To detect HSV-1 specific antibodies of any isotype in macaque serum, two indirect sandwich ELISAs were developed: one using recombinant gD protein, and the other using HSV-1 lysate.

#### Using recombinant gD

Plates were coated with macaque serum for 1–2 h at 37°C. After blocking for 1 h at 37°C with PBS/1% bovine serum albumin (BSA, Sigma), recombinant gD protein (Abcam, Cambridge, UK, 200 ng/well) was added overnight at 4°C. Mouse anti-HSV-1/2 gD antibody (Abcam, 1:500 in PBS/0.1% BSA) was then added for 1 h at 37°C followed by anti-mouse antibody conjugated to horseradish peroxidase (HRP, Promega, Madison, WI, 1:2,000 in PBS/0.1% BSA) for 1 h dark at 37°C. Plates were washed between each step above in PBS/0.5% Tween-20 (Sigma). Signal was developed using 3,3′,5,5′-Tetramethylbenzidine (SureBlue TMB Microwell Peroxidase Substrate Kit, KPL) for 30 min dark at room temperature and the reaction stopped with 0.16 M sulfuric acid. The signal was quantified at 450/650 nm on a plate reader from Molecular Devices (Sunnyvale, CA).

#### Using HSV-1 lysate

A lysate of HSV-1 McKrae was prepared by incubating the stock virus in 10% Triton-X 100 (Bio-Rad, Hercules, CA) and 0.2 mM phenylmethanesulfonyl fluoride (Sigma) for 90 min at room temperature. Lysates were diluted 10 fold in PBS, and aliquots were frozen at −80°C until use. Plates were coated with macaque serum and blocked as for the gD ELISA. To serum-coated, blocked wells, virus lysate (19.4 μg/well total protein) was added and incubated overnight at 4°C. For generation of a lysate standard curve, blank wells were incubated with dilutions of the virus lysate for 2 h at 37°C, washed, and blocked as above. The subsequent detection steps proceeding from addition of mouse anti-HSV-1/2 gD antibody, anti-mouse-HRP, and plate development were all as for the gD ELISA.

### Statistics

All data were graphed using GraphPad Prism version 5.02 for Windows (GraphPad Software, San Diego, CA). Due to the small number of animals included in this pilot study, no statistical analyses could be performed.

## Results

### HSV-1 McKrae infects SIV-infected rhesus macaques orally following mild abrasion

In order to confirm that the intra-oral mucosal epithelia of rhesus macaques are susceptible to productive HSV-1 infection, we exposed buccal and tonsillar tissue explants from three macaques to HSV-1 F *in vitro*. After the decay of the input virus by day 3, HSV-1 grew to a low level in the buccal cultures of two macaques inoculated with the highest viral dose (10^6^ pfu/explant) between days 3 and 7 (Supplementary Figure 1). Tonsil tissue from one of these two macaques also supported virus growth with titers increasing from day 7 to 14. In each tissue that supported virus grown, we were able to inhibit virus growth in another explant from the same macaque with acyclovir (ACV) at 10^6^ pfu/explant. Thus, infection by HSV-1 was possible in macaque oral tissues *in vitro*. Tissues from a third macaque did not support HSV-1 growth.

*In vivo*, we challenged two adult, female, Herpes B negative, SIVmac251 chronically infected rhesus macaques intra-orally with high inocula of HSV-1 F−4 × 10^8^ pfu (EL75) or 1 × 10^9^ pfu (FB90), and followed the animals for 21 days (Supplementary Figure 2, Table 1). However, HSV-1 DNA was detected in oral swab fluid from only one of the two animals (EL75), only at a single time point day 21 post-infection, and only in 1 of 6 replicate nPCR reactions (Supplementary Figure 2). No clear evidence of viral DNA was found in either of the animals' trigeminal ganglia; palatine, pharyngeal, or lingual tonsils; submandibular, retropharyngeal, or cervical lymph nodes; or buccal tissue on day 21 by nPCR performed on a 3 mm punch biopsy of the indicated tissue (0 of 6 replicate reactions positive, Supplementary Figure 2 and data not shown). Moderate decreases were observed in the animals' peripheral CD4^+^ T cell counts during the 21-day follow up period (less than 40%), consistent with their SIV infection status (Table 1).

**Figure 1:**
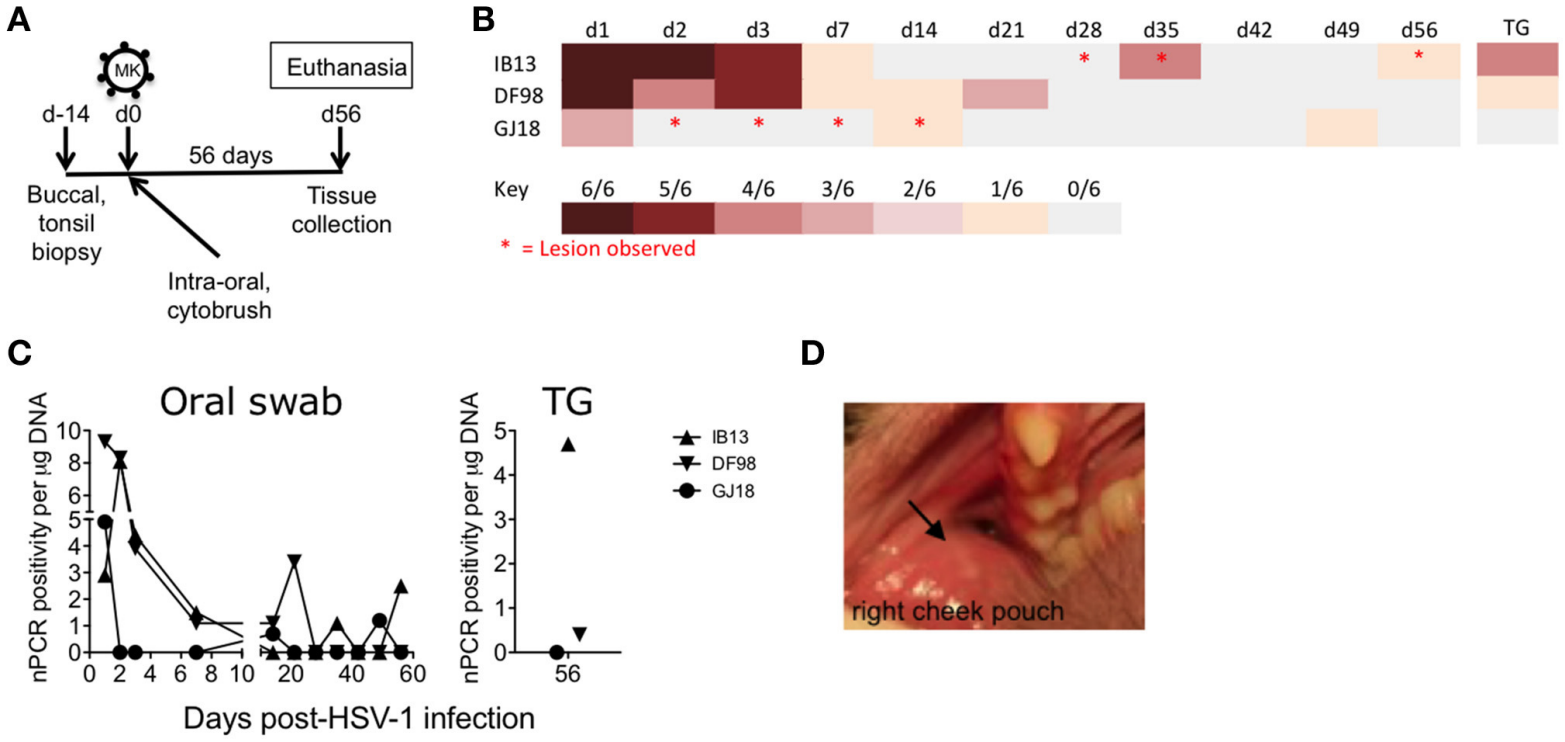
Rhesus macaques infected orally with HSV-1 McKrae exhibit recurrent shedding and oral lesions**. (A)** Schematic showing the study design and tissue collection. Three SIVmac251 chronically infected rhesus macaques were inoculated with 4 × 10^8^ pfu HSV-1 McKrae (MK) by application of virus over all exposed epithelia inside the mouth, including the tonsillar epithelia, after the tissues were first gently abraded with a cytobrush. After 56 days (d56), the animals were euthanized and mucosal, lymphoid, and neural tissues were collected. Buccal and palatine tonsil biopsies were taken 14 days prior to challenge (d-14) and at the time of euthanasia. **(B)** Heat map depicting HSV-1 shedding in DNA extracted from unclarified oral swabs observed over time by nPCR. Each row represents an animal. Each column represents a day (d) post-infection. Trigeminal ganglia (TG) infection was assessed by performing the PCR on explanted cultures of the tissue. Red asterisks indicate oral lesions. **(C)** The percentage of positive PCR reactions of the six reactions performed for each oral swab (left) and TG sample (right) was plotted as a function of the amount of total DNA in the reaction (e.g., 6 of 6 reactions positive for 0.2 μg DNA = 5) **(D)** Lesion 1 mm across (indicated by black arrow) photographed in the right cheek pouch buccal mucosa of IB13 on day 35.

To study if epithelial disruption would enhance infectivity, we abraded the oral mucosal epithelia prior to challenge in three other SIVmac251 chronically infected macaques and used a more virulent strain of HSV-1, McKrae (Figure 1A, Table 1). Upon challenge with the lower of the two doses tested for HSV-1 F (4 × 10^8^ pfu) on the intra-oral epithelia and tonsils, all three macaques (IB13, DF98, and GJ18) became infected with HSV-1 as determined by the presence of HSV-1 US6 DNA in unclarified oral swab fluid by nPCR (Figures 1B,C, Supplementary Figure 3). This nPCR was able to detect one HSV-1 genome per reaction in 4 of 6 replicate reactions performed (Supplementary Figure 4). Specificity of the HSV-1 nPCR products was confirmed by sequencing (Supplementary Figure 4). Primary infection lasted up to 21 days with viral DNA detected at moderate to high levels in oral swabs during the first week and tapering off thereafter (Figures 1B,C). In IB13 and DF98, primary infection exhibited similar kinetics while containment of oral shedding was more rapid in GJ18 (Figures 1B,C). We monitored the animals for 56 days in order to look for reactivation after the resolution of primary infection. IB13 and GJ18 each exhibited two recurrences of low level shedding after periods of no shedding during the follow up period. DF98 also exhibited an increase in shedding on day 21 after shedding had subsided from day 7 through 14 but did not show recurrence thereafter (Figures 1B,C). From time points at which HSV-1 DNA was detected by nPCR, pellets remaining from separation of the clarified oral swab supernatant were subjected to qPCR with a limit of detection below 30 genomes per reaction (Supplementary Figure 4); however, viral DNA was not present above the limit of detection in the pellets (data not shown).

We explanted trigeminal ganglia collected at the time of euthanasia 56 days post-infection for 24 h in an attempt to reactivate viral replication from the neural tissue in which HSV-1 establishes latent infection in humans (Roizman et al., 2007) and obtain a preliminary insight into whether or not HSV-1 infection in these animals might have resulted in latency. HSV-1 DNA was detected by nPCR in the cultured piece of trigeminal ganglia from IB13 (4 of 6 reactions positive) and DF98 (1 of 6 reactions positive; Figures 1B,C, Supplementary Figure 3). However, it was not detected in a piece of the tissue from which the PCR was performed without explanting (0 of 6 reactions positive, data not shown). No HSV-1 DNA was detected in trigeminal ganglia from GJ18 (Figures 1B,C, Supplementary Figure 3).

To determine if lesions accompanied virus shedding, we monitored the clinical consequences of oral HSV-1 infection in HSV-1/SIV-co-infected macaques on days 1, 2, 3, 7, 14, 21, 28, 35, 42, 49, and 56 (Table 1). Oral epithelial lesions were observed in buccal mucosa of IB13 and GJ18, but not DF98 (Figures 1B,D). Lesions grew from pinpoint ulcers to 1–2 mm raised vesicles/papules and then receded. IB13, the macaque with the most extended shedding and strongest evidence of neuronal infection, was the only macaque with oral lesions in the post-primary infection phase, one evident in the right buccal cheek pouch from day 28 to 35 (Figure 1D), and another also in the right buccal cheek pouch on day 56. Notably, both lesions coincided with HSV-1 shedding in oral swab fluid (day 35 and day 56, respectively, Figure 1B). In addition, IB13 had periodontal hemorrhage on day 1, enlarged axillary lymph nodes on days 3, 7, and 14, and high neutrophil and total white blood cell count on days 28 and 42 (Table 1). IB13 also suffered poor appetite from day 7 to 14 and a bloody nasal discharge on day 28. For DF98, all parameters were within normal limits throughout the study (Table 1). For GJ18, parameters were also within normal limits but this macaque experienced extended ulceration during the study: in the gingiva (bilateral pinpoint ulcers on day 2), left dorsal cheek pouch (raised ulcers/papules observed on days 3 and 7), and upper left labial mucosa (day 14), as well as mild SIV-related periodontal hemorrhage in the right upper dental arcade pre-inoculation (Table 1). The lesion on day 14 coincided with shedding (Figure 1B). Overall, these data indicate that intra-oral mucosal infection with HSV-1 in rhesus macaques is possible *in vivo* following mild tissue abrasion and that mucosal infection may lead to a low-level neuronal infection and recurrent shedding associated with lesions.

### Oral HSV-1 infection may modulate SIV viral load in blood and oral tissues of SIV-infected macaques

**Figure 2:**
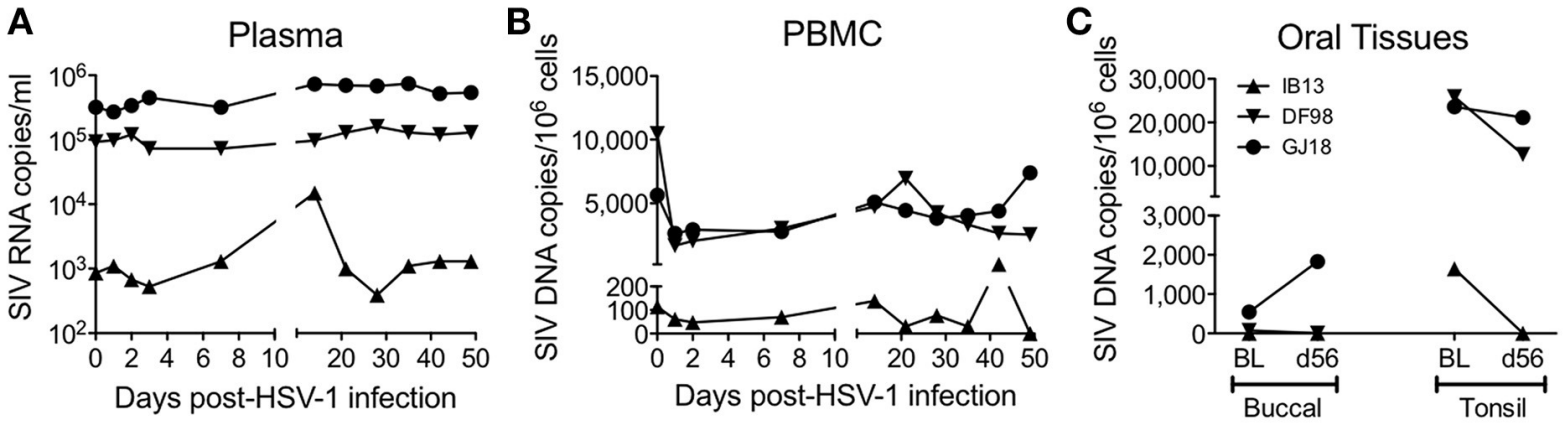
Oral HSV-1 infection may modulate SIV viral load in co-infected macaques. SIV levels were documented over time following HSV-1 infection. **(A)** SIV RNA copies/ml in plasma. SIV DNA copies/million cells in **(B)** PBMCs and **(C)** oral tissues. In **(A,B)**, measurements were made over time before and following HSV-1 infection. In **(C)**, SIV DNA was measured in buccal and palatine tonsil tissue biopsies at the baseline (BL) 14 days prior to HSV-1 infection and on day 56 (d56) at the time of euthanasia.

At baseline, IB13 had low SIV plasma viral load (<10^3^ copies/ml) in contrast to DF98 and GJ18, which both had high plasma viral load (~10^5^ copies/ml) (Figure 2A, Table 1). IB13—the animal with the most evidence of oral lesions, shedding, and ganglia infection—experienced a transient 17-fold increase in SIV viral load during the first 2 weeks of infection before dropping again to 1.9-fold above baseline by day 56 (Figure 2A). DF98 and GJ18 experienced little if any increase in plasma SIV RNA during the same time period: only in GJ18 was a 2.3-fold increase seen that dropped to 1.6-fold above baseline by day 56 (Figure 2A). Consistent with the overall unperturbed levels of SIV RNA in plasma, no substantial changes in peripheral blood CD4^+^ T cell count were observed for any of the three macaques (Table 1). IB13 and GJ18 had slightly increased and unchanged CD4^+^ T cell counts, respectively. Only in DF98 was there a decrease, which was <40% and on par with the macaques inoculated with HSV-1 F.

Similar to the levels of SIV RNA in plasma, the levels of SIV DNA in PBMCs were lowest for IB13 and higher for DF98 and GJ18 at baseline and throughout the study (Figure 2B). In contrast to the plasma SIV RNA levels, however, PBMC SIV DNA levels fell in all macaques within the first day post-infection by 1.9 (IB13), 6.3 (DF98), and 2.1 (GJ18) fold, recovering over the ensuing weeks to peak at day 14 (IB13 and GJ18) or day 21 (DF98) at levels close to baseline where they stayed for the remainder of the study (Figure 2B). Oral tissue SIV DNA levels also paralleled the blood levels at baseline, but oral tissue viral load was markedly affected following HSV-1 infection (Figure 2C, Table 1). In both IB13 and DF98, SIV DNA levels in the palatine tonsils decreased more than 2-fold between baseline and day 56 post-infection and also decreased albeit slightly (1.1-fold) in GJ18 (Figure 2C, Table 1). During this same time period, SIV DNA levels increased in the buccal mucosa of GJ18, the only macaque with detectable SIV DNA in buccal tissue at baseline, by 3.4-fold (Figure 2C, Table 1). Thus, oral HSV-1 infection transiently modulated SIV viremia in the local mucosal and lymphoid tissues as well as the systemic circulation.

### SIV-infected macaques inoculated orally with HSV-1 McKrae mount a rapid inflammatory response locally and systemically without developing a strong HSV-1-specific adaptive response

**Figure 3:**
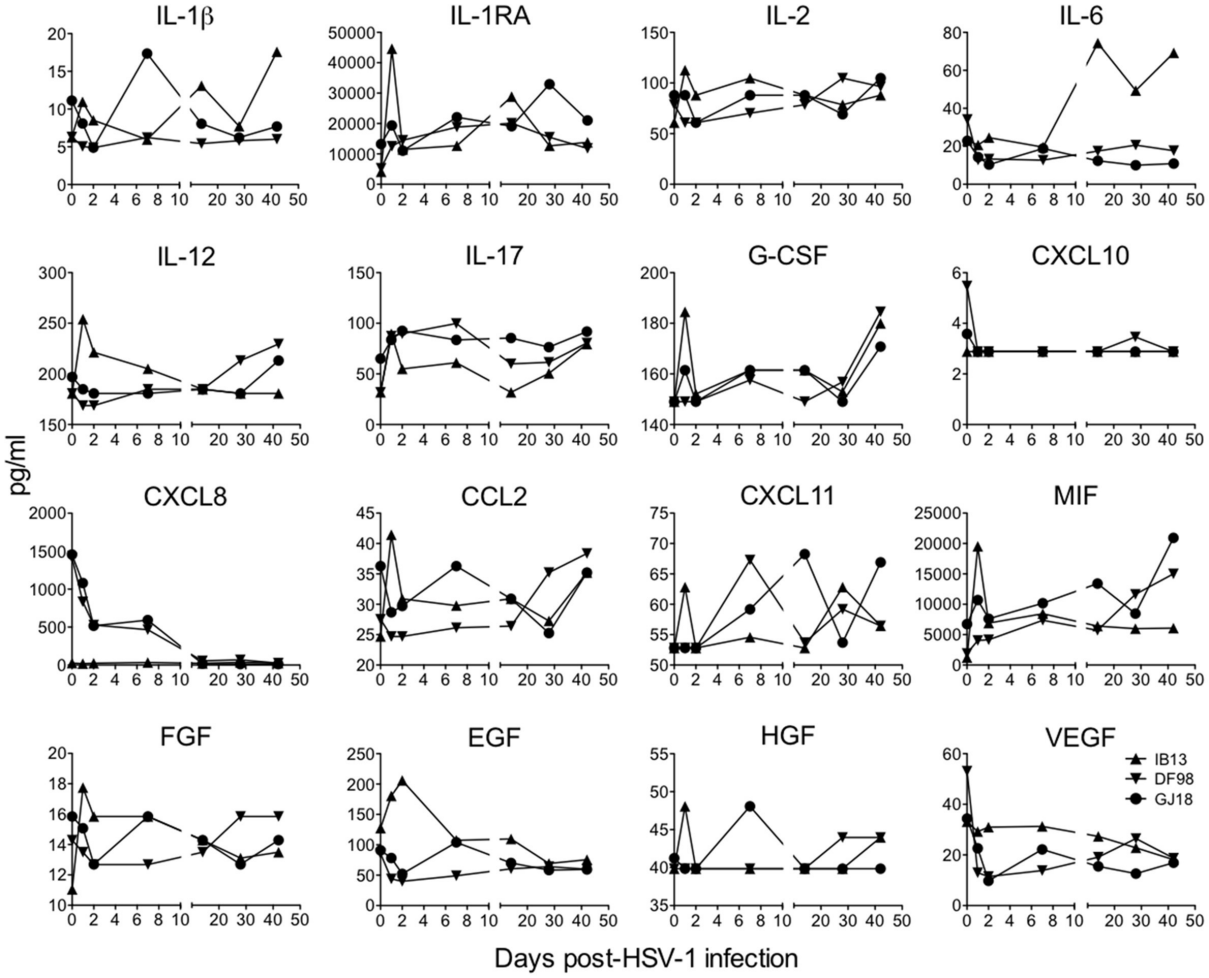
Oral HSV-1 infection elicits a low level, inconsistent pro-inflammatory response in SIV-infected macaques. The concentration of soluble mediators (mean of duplicates) in each animal's clarified oral swab fluid is shown over time following HSV-1 infection. Only those analytes in which the concentrations fell above the lowest standard are shown and concentrations that dipped down below the lowest standard are plotted as the lowest standard value. Plasma cytokine levels are presented in Supplementary Figure 5.

To appreciate the immune responses following oral HSV-1 infection in rhesus macaques, we first measured soluble mediators in clarified oral swabs (Figure 3). A local immune response was evident by day 1 post-infection in IB13. Though modest in magnitude, the response was broad in scope, characterized by transient increases greater than 1.5-fold in several pro-inflammatory cytokines, chemoattractants, and growth factors (IL-1β, IL-1RA, IL-2, IL-12, IL-17, CCL2, MIF, and FGF), as well as smaller or delayed increases in others (CXCL11, G-CSF, EGF, and HGF). The increase in IL-6 was delayed until day 14, and some cytokines (e.g., CXCL8) were undetectable. DF98 and GJ18, on the other hand, exhibited early decreases in most of the same factors in the oral swabs with more than 1.5-fold reductions in IL-6, CXCL10, CXCL8, EGF, and VEGF, and smaller decreases in IL-1β, IL-2, IL-12, CCL2, and FGF. The only immune mediators that initially increased in oral swabs from these two macaques were IL-1RA, IL-17, and MIF (all greater than 1.5-fold). By day 2 to 7, early changes in levels of immune mediators resolved. Around day 21, when primary HSV-1 shedding was subsiding, a second wave of secretion followed for some factors, including G-CSF, IL-6, IL-12, CCL2, MIF, and IL-17.

Responses were distinct in plasma from those in oral swabs (Supplementary Figure 5). Only GJ18 experienced any systemic pro-inflammatory response with transiently increased plasma concentrations of IL-12, CXCL8, and MIF by day 1 to 2 post-infection that rapidly resolved to baseline and later increases in CCL2 and CCL11. IB13 and DF98 both exhibited early mild decreases or no change in all of the factors examined in plasma. Thus overall, local and systemic innate inflammatory responses to HSV-1 infection in these macaques were low magnitude and variable.

**Figure 4:**
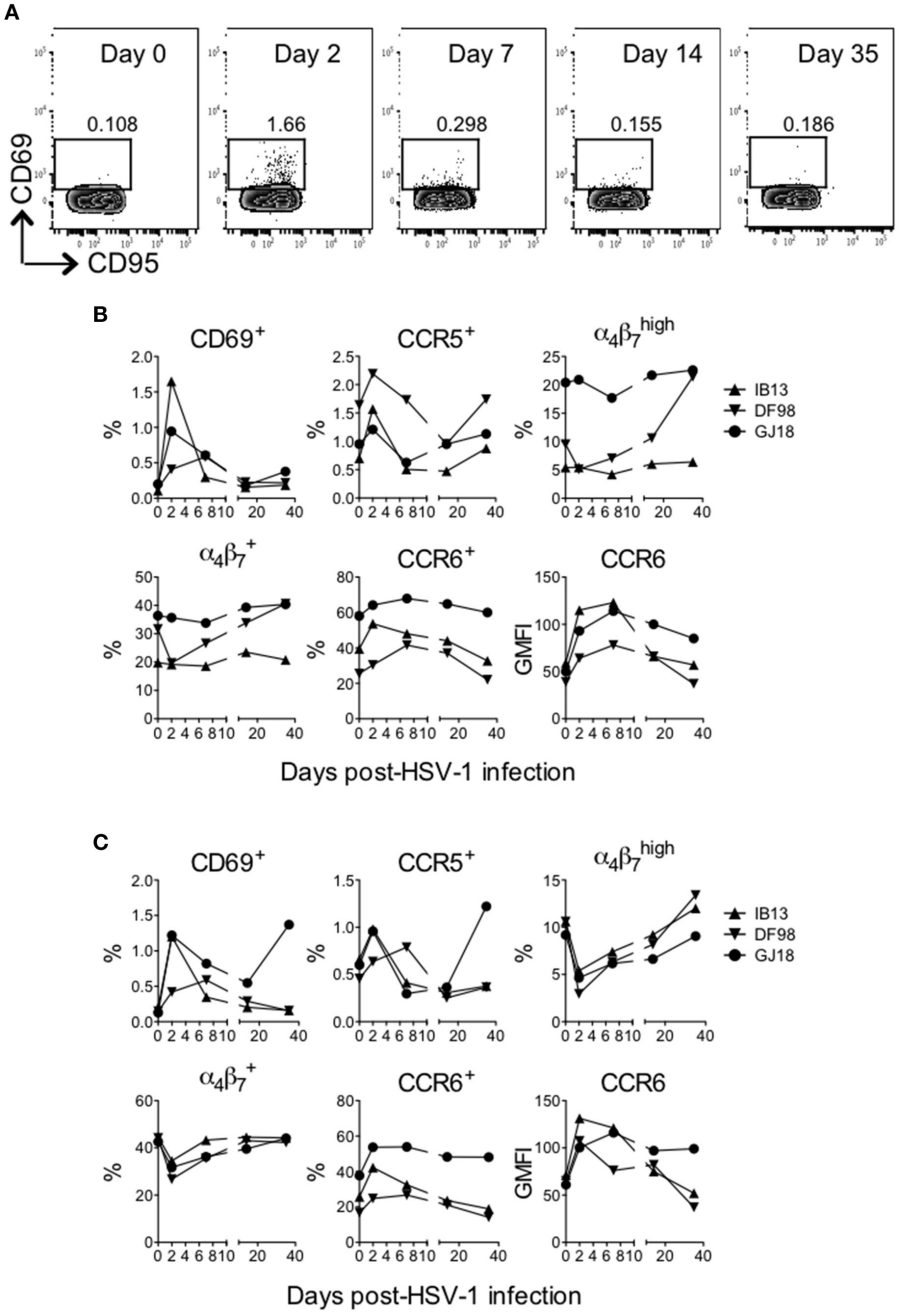
HSV-1 infection rapidly activates T cells in blood of SIV-infected rhesus macaques. The frequency of live (LIVE/DEAD Aqua negative) CD3^+^CD4^+^ and CD3^+^CD8^+^ cells in blood was monitored over time after HSV-1 infection. **(A)** Gating strategy for the CD69^+^ cells within the CD3^+^CD4^+^ T cell gate shown for IB13. CD95 is on the x-axis and CD69 is on the y-axis. By day 2, activation was observed predominantly of the memory (CD95^+^) population. The frequency of **(B)** CD4^+^ and **(C)** CD8^+^ T cells expressing the indicated activation and cell subset markers or the geometric mean fluorescence intensity (GMFI) of indicated markers are shown over time.

HSV infection in humans is largely controlled by the T cell response (Ouwendijk et al., 2013), and CD4^+^ T cells persisting in anogenital HSV-2 lesion sites possess characteristics that render them highly susceptible to HIV infection, such as an activated (e.g., CD69^+^) CCR5^+^ or $\alpha_{4}\beta_{7}^{\text{high}}$ phenotype (Zhu et al., 2009; Martinelli et al., 2011; Goode et al., 2014; Shannon et al., 2014). Thus, we monitored changes in peripheral blood CD4^+^ and CD8^+^ T cell phenotype during the first month following oral HSV-1 inoculation. Oral HSV-1 infection in SIV-infected macaques drove rapid activation of T cells in blood. Within the CD4^+^ and CD8^+^ T cell compartments, the frequency of cells expressing CD69 and CCR5 increased (up to 14-fold) within 2 days of infection before declining again by day 7 (Figure 4, Supplementary Figure 6). The frequency of CD4^+^ and CD8^+^ T cells expressing CCR6, a chemokine receptor that directs homing of lymphocytes in response to inflammation and marks Th17 cells, which are also highly susceptible to HIV and SIV infection (Cecchinato et al., 2008; Alvarez et al., 2013), also increased during the first 7 days before declining to baseline levels by day 35. While the overall CCR6^+^ population increased only slightly (up to 1.3-fold), cells expressing higher levels of CCR6 (see gating strategy in Supplementary Figure 6) increased more as evidenced by the greater increase in geometric mean fluorescence (GMFI) of CCR6 (up to 2.8 and 1.9-fold on CD4^+^ and CD8^+^ T cells, respectively, Figures 4B,C). In contrast to CD69, CCR5, and CCR6, no consistent change was seen in expression of α_4_β_7_ on CD4^+^ T cells. Only in DF98 was a 4.1-fold increase evident in the $\alpha_{4}\beta_{7}^{\text{high}}$ population from day 2 to day 35 after an initial decrease. On CD8^+^ T cells, an early decrease in the frequency of α_4_β_7_-expressing cells was evident in all macaques (up to 3.6-fold for $\alpha_{4}\beta_{7}^{\text{high}}$), with the cell frequency recovering to or above baseline by day 35. Most of the cells expressing activation and homing molecules at the height of the response were contained within the memory (CD95^+^) fraction, especially for IB13 (Figure 4A, Supplementary Figure 6); however, increased cell subset frequencies were not due to a general increase in CD95^+^ T cells (data not shown). Overall, IB13 and GJ18 responded more robustly than DF98. Together, these results suggest that oral HSV-1 infection in macaques rapidly activates peripheral CD4^+^ and CD8^+^ T cells and either alters the T cells' expression of chemokine receptors and integrins important for tissue homing and HIV infection or mobilizes specific cell subsets into or out of the blood.

**Figure 5:**
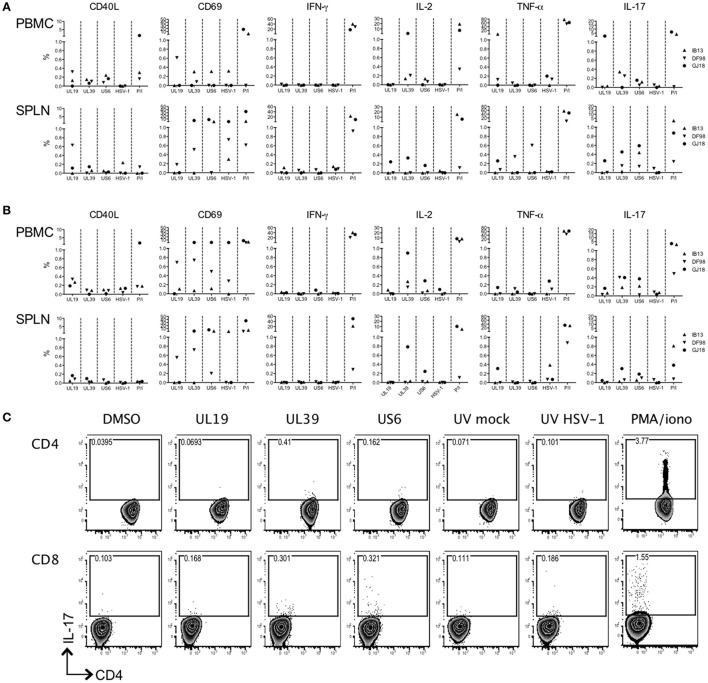
HSV-1-specific T cell induction is low and uncharacteristic in HSV-1/SIV co-infected macaques. PBMCs and splenocytes (SPLN) collected at the time of euthanasia 56 days post-HSV-1 infection were stimulated with pools of HSV-derived peptides (vs. DMSO negative control) spanning three immunogenic open reading frames (UL19, UL39, and US6) or with an HSV-1 lysate (vs. mock lystate negative control). PMA/ionomycin (P/I) served as the positive control. HSV-specific responses were defined by subtracting the relevant background from the specific response. The **(A)** CD4^+^ and **(B)** CD8^+^ responding cells within the PBMCs and splenocytes are shown. **(C)** Gating strategy for the IL-17 secreting PBMCs within the CD3^+^CD4^+^ and CD3^+^CD8^+^ T cell gates of IB13. CD4 is on the x-axis and IL-17A is on the y-axis.

Although past studies of macaque vaginal HSV-2 infection have seen little development of HSV-specific T cell responses during the first several months of infection (Crostarosa et al., 2009; Hsu et al., 2014), the rapid activation of T cells in blood suggested that oral HSV-1 infection might be more immunogenic. We assessed the development of an HSV-1 specific T cell response by intracellular cytokine staining flow cytometry on PBMCs and splenocytes from the macaques after 56 days of infection. The stimuli induced varying levels of activation in macaque cells (CD40L and CD69 expression) (Figures 5A,B). However, while cells from IB13 and GJ18 (less so from DF98) were responsive to PMA/iono stimulation, they were overall poorly responsive to HSV antigens and displayed an uncharacteristic response. Neither blood nor spleen CD4^+^ or CD8^+^ T cells secreted much IFN-γ in response to peptide or viral lysate stimulation (Figures 5A,B). CD4^+^ and CD8^+^ T cells in blood (especially of GJ18) produced some IL-2 in response to peptides, particularly UL39, but little of this was in cells expressing CD69 as a marker for activation (Supplementary Figure 7). Blood and spleen cells from all three macaques secreted a small amount of TNF-α in response to variable antigens, and some of this was in cells co-expressing CD69 (Figures 5A,B, Supplementary Figure 7), but the background on relevant control-stimulated cells was high (Supplementary Figure 8). Because of the observed shift in CCR6-expressing T cells in blood during the study, we included antibody to IL-17A in the staining panel even though HSV-specific cells do not characteristically produce IL-17 in humans. Indeed, we detected CD4^+^ and CD8^+^ cells in blood and spleen that secreted IL-17A, and some of these cells co-expressed CD69 (Figure 5C, Supplementary Figure 6). Given the low frequency of HSV-specific cells producing even a single cytokine, multi-cytokine producing HSV-specific T cells were not quantified. These data indicate that by day 56 post-infection, a low level HSV-specific T cell response was evident in HSV-1/SIV co-infected macaques but that the responding cells were atypical; they were poorly activated and secreted IL-17 but not IFN-γ. Opposed to their extent of HSV-1 infection, GJ18 was the greatest overall responder, and IB13 was the poorest.

**Figure 6:**
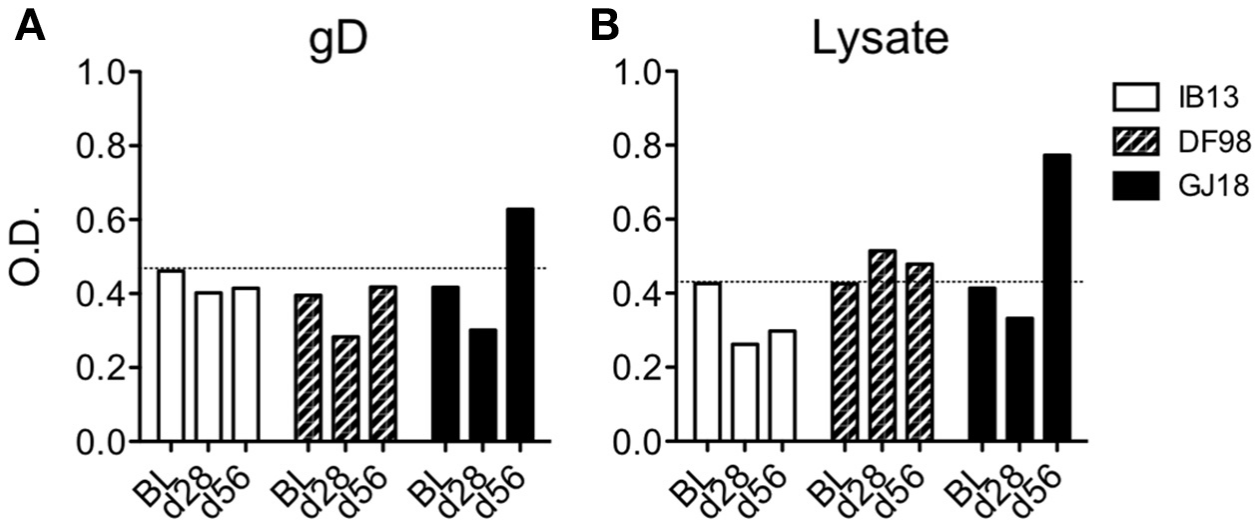
Figure 6. HSV-1 seroconversion is low level and late in HSV-1/SIV co-infected macaques. HSV-specific antibodies were measured in serum by indirect sandwich ELISA at day 28 (d28) and day 56 (d56) post-HSV-1 infection vs the baseline (BL, 14 days before HSV-1 infection). Either **(A)** recombinant gD protein or **(B)** lysate of HSV-1 McKrae was titrated onto serum-coated plates to detect HSV-specific antibodies independent of isotype in the serum. The optical density (O.D.) of the signal is reported. In both **(A,B)**, the dotted line represents the mean plus one standard deviation of the baseline readings of the three animals.

In addition to measuring T cell responses, we sought to determine whether or not the macaques seroconverted. So as to not limit the detection by expected antibody isotype, we developed an ELISA based on coating plates with serum rather than titrating serum on plates coated with HSV antigen. Using either recombinant gD protein or a lysate of HSV-1 McKrae to detect anti-HSV-1 antibodies in the sera, we found that only GJ18 exhibited a positive antibody response above the background and that it was only just detectable at day 56, not day 28 (Figure 6). The response was somewhat greater when we used lysate containing all HSV-1 proteins than gD alone.

## Discussion

HSV-1 and−2 are ubiquitous human pathogens for which we have no cure and no licensed vaccine. While genital HSV-2 infection is known to exacerbate the risk of acquiring HIV infection by several fold (Stamm et al., 1988; Freeman et al., 2006) and has been associated with higher HIV viral loads in co-infected subjects compared to HSV-2 uninfected subjects (Barnabas and Celum, 2012), no studies have examined the influence of HSV-1 infection in the oral mucosa on oral HIV transmission or HIV disease. This is due at least in part to the absence of a relevant animal model for studying oral HSV-1 co-infection in the context of HIV infection. Only one recent study described oral HSV-1 infection in rhesus macaques (Fan et al., 2017), and this was in the absence of SIV. The majority of NHP studies of HSV-1 infection have used new world NHPs, with highly pathogenic outcomes (Kaufman, 1963; Katzin et al., 1967; Varnell et al., 1987, 1995; Rootman et al., 1990; Hunter et al., 1999; Deisboeck et al., 2003). To facilitate investigation of the HSV-1/SIV relationship, we needed first to demonstrate the feasibility of the co-infection as well as to explore the resulting immunological changes. Herein we report that experimental oral infection with HSV-1 in SIV-infected rhesus macaques produced low-level viral replication with limited and atypical immune responses. Oral HSV-1 infection affected SIV viremia and immune cell subsets at least transiently, suggesting that this otherwise innocuous oral infection may contribute to HIV disease. However, further studies with more animals and clear control groups for the effects of each virus on the other will be needed to verify and expand these findings.

Atraumatic inoculation with HSV-1 F did not lead to productive infection in macaques. This is perhaps not surprising given the low level of viral replication observed in buccal mucosa explant cultures exposed to HSV-1 F though SIV infection and resulting immunosuppression might have been expected to facilitate *in vivo* infection. However, applying the highly virulent HSV-1 strain, McKrae, to gently abraded oral epithelia resulted in infection of all three SIV-infected macaques. HSV-1 DNA was repeatedly detected in oral swabs, and although there was the potential to detect the inoculum, all three macaques experienced increased viral HSV DNA levels in oral swabs after shedding cessation or reduction, indicating *de novo* virus replication *in vivo*. Mouse models of HSV infection routinely create a more permissive environment to virus replication by thinning the epithelium with depot medroxyprogesterone acetate (vaginal HSV infection) or scarifying the corneas (ocular HSV infection). Prior comparisons of HSV-1 F and HSV-1 McKrae in mouse models have demonstrated that McKrae is more pathogenic and requires a lower dose to induce morbidity and mortality in mice than HSV-1 F (Dix et al., 1983; Wang et al., 2013). McKrae also does not require scarification for ocular infection in tree shews (Li et al., 2016). Due to the limitations of this pilot experiment, we were unable to test oral inoculation of macaques with McKrae in absence of abrasion to determine whether it would be possible. Recently, we modeled vaginal HSV-1 McKrae infection in rhesus macaques and found that infection occurred only after abrasion (Aravantinou et al., 2017). In the only other description of oral HSV-1 infection of macaques, lip scarification was utilized to enable infection with strain F (Fan et al., 2017). Thus, although it remains possible that the difference between our first and second oral HSV-1 infection attempts was the strain of virus used, it is likely that an enhanced mode of virus introduction is needed to facilitate infection in rhesus macaques.

Primary oral HSV-1 infection in macaques consisted of shedding for 7–21 days with the greatest shedding during the first week, which is similar to the ~12 day primary infection period in humans (Wald and Corey, 2007). Shedding in macaques produced low amounts of HSV-1 DNA in mucosal fluid. Unclarified swabs contained sufficient US6 DNA for detection in multiple replicate nPCR reactions and provided an indication of relative levels of shedding over time. However, there was not sufficient viral DNA in the swab pellets for detection of UL27 by qPCR. This may be due to the type of sample (the pellet may contain less HSV-1 DNA than the unclarified swab) or may be due to the difference in sensitivity between the nPCR and the qPCR. The overall low levels of HSV-1 DNA present in swabs from macaques may highlight a divergence from human infection in which high copy numbers of HSV can often be detected at times of shedding.

Despite the low levels of viral DNA detected, we were able to observe recurrence during 2 months of follow up. In immunocomptent and HIV-infected adults, oral HSV-1 reactivation is not a common event and is short lived, on the order of under 12 h (Griffin et al., 2008; Mark et al., 2008, 2010). More frequent sampling would likely have increased detection in our study but was not possible for reasons of minimizing sedation and animal handling stress. Lesions developed during both the primary and recurrent phases of infection in some cases in connection with shedding, and lesions were of similar size to those reported in humans (Sacks et al., 2004). However, while recurrent oral HSV-1 infection in humans usually produces lesions on the lips and outer thin skin of the mouth, all of the lesions we observed in the macaques were intra-oral on the buccal and gingival mucosa. This is most likely related to the strictly intra-oral inoculation procedure but could alternatively be a fundamental difference in the course of infection in macaques vs. humans. Intra-oral inoculation was performed to avoid initial transmission of the virus from mouth to eye by macaques touching their faces and the possibility of keratitis as found in new world NHPs (Varnell et al., 1987, 1995; Rootman et al., 1990). Though it is possible that the initial ulcerations and pro-inflammatory responses were related to the abrasion, it is unlikely that the recurrent lesions in IB13 were related to abrasion in absence of HSV-1 infection. IB13 exhibited the most robust HSV-1 infection characterized by the greatest acute shedding, the most recurrence associated with lesions, and the most HSV-1 DNA detected in the trigeminal ganglia. DF98 and GJ18 had less shedding recurrence and little to no HSV-1 DNA detected in the trigeminal ganglia. These two macaques also had SIV plasma viral load at least 2 logs higher than IB13 and much lower CD4 counts, close to or below the 200 cells/ml cut off defining AIDS. In modeling vaginal HSV-1 infection in macaques, we also observed the greatest vaginal HSV-1 shedding in the macaque with undetectable SHIV viral load (Aravantinou et al., 2017). Immunosuppression has been linked with asymptomatic rather than symptomatic HSV shedding in humans (Sacks et al., 2004) and may have contributed to differences in shedding observed herein. In addition, it is possible that a type I interferon response elicited by SIV infection could have limited HSV-1 infection.

In humans, HSV recurrence is facilitated by latency and reactivation. Consistent with the greatest recurrence of mucosal shedding and lesions in IB13 and mirroring the direct relationship between recurrence and extent of neural infection in mice (Sawtell, 1998; Sacks et al., 2004), IB13 also had the most HSV-1 DNA detected in the trigeminal ganglia. However, HSV-1 DNA was detected only after explanting the ganglia and not in uncultured tissue. It is possible that the piece of tissue from which PCR was performed without culturing simply did not contain cell bodies housing the viral DNA or that the amount of HSV-1 DNA in the ganglia was quite low and that reactivation of viral replication through culture increased the signal above the level of detection of the assay. Either way, it is notable that neural infection was low when it occurred and did not always appear to occur, as in the case of GJ18. However, the 24-h period allowed for reactivation *in vitro* may not have been long enough to achieve reactivation to detectable levels in the case of low-level ganglia infection as reactivation *in vitro* is assay and time dependent (Doll and Sawtell, 2017). In addition to being associated with greater HSV-1 shedding, neural infection level trended with lower SIV viral load. Interestingly in macaques orally infected with HSV-1 in absence of SIV, trigeminal ganglia harvested a year post-infection contained HSV-1 DNA detectable without explanting (Fan et al., 2017). Whether detection of neural infection is related predominantly to the method of inoculation, the amount of HSV shedding, the duration of time between infection and ganglia sampling, the SIV-infection and immunosuppression status of the animals, or other factors requires further exploration.

Genital HSV-2 infection is associated with increased frequencies of T cells with an HIV susceptibility profile (e.g., activated and CCR5 or α_4_β_7_-expressing) in human blood and mucosal tissue (Zhu et al., 2009; Shannon et al., 2014), and this has also been described in macaques (Martinelli et al., 2011; Goode et al., 2014). Here, we found a similar result in the blood of orally HSV-1 infected macaques. CD69 and CCR5 were upregulated early after infection on CD4^+^ T cells in all three animals, and α_4_β_7_ expression also increased in DF98 over time despite an initial drop.

IB13 experienced the largest increase in plasma SIV viral load after oral HSV-1 infection. This could have been simply related to the fact that viral load was low enough at study inception for an increase to be detectable or could be linked with the more pronounced HSV-1 infection in IB13 (which may also have been related to this animal's lower SIV viral load). GJ18 and DF98, which both began the study with high SIV plasma viral load, experienced little if any increase in plasma SIV RNA after HSV-1 infection. Effects of HSV-1 infection on SIV infection were apparent in PBMC and oral tissue SIV DNA as well as in plasma SIV RNA. The drop in SIV DNA level in PBMCs on day 1 post infection suggests that cells harboring SIV DNA may have emigrated out of the blood (potentially toward the oral mucosa) in response to HSV-1 infection or become activated, disrupting latency and resulting in destruction of the cells. Supporting the notion that HSV-1 infection may recruit SIV-infected or SIV target cells to the mucosa or reactivate SIV replication, we observed a decrease in SIV DNA in the palatine tonsils of all three macaques with a concomitant increase in SIV DNA in the buccal tissue of GJ18, the animal with highest SIV viral load and the only animal with detectable buccal SIV DNA at the baseline. However, we were unable to phenotype immune cells in the mucosa to investigate these possibilities, and no HSV-1 uninfected controls were available for cross-sectional analysis. Future work is needed to tease out whether the changes in SIV levels reflect local viral clearance, trafficking or destruction of the SIV-infected cells, or reactivated SIV replication from latency in newly activated cells and whether these changes have implications for oral SIV disease.

Along with the most robust HSV-1 infection, IB13 was the only macaque to exhibit a local pro-inflammatory response including secretion of IL-1, IL-12, and IL-6. However, even these characteristic responses were low level (IL-12) or delayed (IL-1 and IL-6), and no IFN**-**γ could be detected in oral swabs. Nonetheless, IB13 also exhibited the greatest acute increase in CD69 expression on peripheral blood T cells. DF98 exhibited the least local response, the least acute T cell activation, and also the most protracted primary infection shedding, suggesting a possible link between the acute immune effects and viral control in this model. The direct relationship between the pro-inflammatory response, the oral HSV-1 shedding, and the change in SIV viral load in IB13 suggests that HSV-1 infection (and not the abrasion during inoculation) was responsible. However, future studies with a true control group (macaques subjected to oral mucosa abrasion and sham inoculation) will be needed to confirm that indeed the pro-inflammatory response and SIV viremia are not aggravated by cytobrushing alone. The decline in CXCL8 in DF98 and GJ18 was similar to that observed following vaginal HSV-1 infection in macaques (Aravantinou et al., 2017) and needs further study. Increased frequency of CCR6-expressing T cells, particularly CD4^+^ T cells, was a novel finding in these macaques. Whether or not these cells represented Th17-like cells was not assessed; however the detection of cells secreting IL-17 in response to HSV-1 stimulation on day 56, suggests the possibility that HSV infection in macaques may trigger a Th17-like response.

To afford the best chance of detecting virus-specific CD4^+^ and CD8^+^ T cells, we stimulated with virus lysate and peptide pools covering some of the proteins known to be immunodominant in humans (Ouwendijk et al., 2013). Still, despite the rapid systemic T cell activation, we detected only low-level responses with no IFN-γ secretion in poorly activated cells. In human infection, HSV-specific T cells secrete IFN-γ along with other cytokines (Ouwendijk et al., 2013). Past studies of macaque vaginal HSV-2 infection in which the macaques shed HSV-2 sporadically for up to a year or more also saw little development of HSV-specific T cell responses during the first several months of infection (Crostarosa et al., 2009; Hsu et al., 2014). However in those studies, IFN-γ was indeed produced by blood and lymph node cells several months post-infection even in animals co-infected with SIV (Crostarosa et al., 2009; Hsu et al., 2014). Macaques vaccinated with HSV immunogens in recently reported studies also readily developed T cell responses including IFN-γ production (Awasthi et al., 2017; Stanfield et al., 2017). In this study, we were limited by having to use cryopreserved cells for stimulation and recovered a small proportion of viable cells. This may have contributed to the poor responses observed though it is unlikely to have specifically affected IFN-γ over other cytokines. It is also possible that HSV-specific T cells may have been concentrated in the oral mucosa where we did not look, but at the end of the study, we did not observe any increase in IFN-γ (or IL-2 or TNF-α) levels in oral swabs. We did, however, observe increasing IL-17 levels in swabs from IB13. Additional work is needed to clarify these results on the T cell response, which could have been influenced by the cell condition, the time post-infection, and the anatomic compartment, as well as by the immunobiology of HSV infection in macaques.

Seroconversion was also not pronounced. We developed two ELISA assays capable of detecting antibodies of any isotype that could react with either McKrae virus lysate or gD protein. Yet only GJ18—the macaque with highest SIV viral load in blood and oral tissues, least HSV-1 shedding, and absence of HSV-1 DNA in ganglia—mounted an antibody response over the course of the 8 week study. We have not yet elucidated whether these were IgM or IgG. Using commercially available reagents designed for detecting IgG antibodies in humans, we have also previously found only low level or absent ELISA responses in HSV-2 infected animals (Crostarosa et al., 2009; Hsu et al., 2014). However, rhesus macaques are indeed able to mount antibody (including IgG) as well as T cell responses to HSV-2-derived immunogens (Awasthi et al., 2017). More needs to be learned about the antibody response to HSV infection in NHPs.

The difference between HSV-1 infection in rhesus macaques and that in new world NHP models cannot be overstated. In fact, there has been a prevailing perception in the field that HSV-1 infection will cause encephalitis in rhesus macaques because it proceeds this way in new world NHPs. However, an additional body of research supports the concept that macaques may be inherently resistant to HSV infection (Nahmias et al., 1970; London et al., 1974; Juan-Sallés et al., 1997; Reszka et al., 2010). Much more research is needed to assess to what degree the variable *in vivo* virulence in NHP models is a function of inoculation (the route, dose, presence, or absence of tissue trauma), or the species and sub-species. That vaginal HSV-2 infection in macaques also requires high inocula, results in limited shedding, and has been described as a self-limiting infection (Awasthi et al., 2017) further suggest the need for an improved understanding of how HSV infections in NHPs differ from those infections in humans. A natural endemic alphaherpesvirus of old world NHPs, herpes B virus, is not pathogenic in macaques, and comparative studies could prove enlightening. However, the high neurovirulence of herpes B virus in humans and the resulting BSL4 status of this organism restrict its capabilities for study. A better understanding of HSV infection in macaques and why it is not so pathogenic may enable the elucidation of new targets for prevention and therapy for human infection. For a model of oral HSV-1 infection in the context of SIV infection to be useful in improving our understanding of the relationship between these infections, it will need to be better characterized to acknowledge its limitations and refined to mimic more closely the disease course in humans. Further development of the oral HSV-1 and HSV-1/SIV infection models in macaques may allow for the testing of prevention and treatment strategies for both viruses and enable us to appreciate the intersection of HSV-1 and HIV infections in the oral mucosa.

## Author contributions

MA, OM, GC, JK, and IF: processed macaque samples, performed assays, and contributed to the manuscript; BG, JB, and AG: carried out macaque experiments. MS, LJ, and DMK: provided reagents; JL: performed SIV viral load testing, assisted in data interpretation, and revised the manuscript; MS, DMK, AG, JB, BG, NT, and EM: assisted in experimental design and data interpretation and revised the manuscript; ND designed the study, analyzed the data, and wrote the manuscript.

### Conflict of interest statement

The authors declare that the research was conducted in the absence of any commercial or financial relationships that could be construed as a potential conflict of interest.

## Supplementary material

Supplementary Figure 1 Macaque oral tissues are susceptible to low-level HSV-1 F infection *in vitro*. Buccal and tonsil explants (3 × 3 × 3 mm) were exposed to HSV-1 F at 10^3^, 10^4^, 10^5^, or 10^6^ pfu/explant for 18 h. Explants exposed to 10^6^ pfu/piece were cultured in the absence or presence of acyclovir (ACV). After extensive washing, tissues were cultured for 14 days. Supernatants collected over time were assayed for the presence of infectious virus by plaque assay on Vero cells. Tissues from three macaques were inoculated in triplicate for each tissue type and virus dose. The result of replicate cultures from each animal at each viral dose is shown (mean ± SEM). No virus grew in any culture containing ACV. Data from infection with 10^3^ pfu were omitted from the graphs as no infection was observed.

Supplementary Figure 2 Atraumatic intra-oral inoculation with HSV-1 F does not reliably infect rhesus macaques *in vivo*. **(A)** Study schematic. Two SIVmac251 chronically infected rhesus macaques were inoculated atraumatically intra-orally with 4 × 10^8^ (EL75) or 1 × 10^9^ (FB90) pfu HSV-1 F. Virus was applied over all exposed epithelia inside the mouth, including the tonsillar epithelia. After 21 days (d21), the animals were euthanized and multiple mucosal, lymphoid, and neural tissues were collected. **(B)** To monitor HSV-1 shedding, nested PCR in US6 (six replicate reactions per sample) was performed on DNA extracted from unclarified oral swabs collected at the baseline (BL) and on days 3, 7, 14, and 21 post-HSV-1 infection. Internal products were separated on agarose gels and the 137 base pair product was visualized. The PCR product showing amplification from EL75 on day 21 was sequenced, confirming the product was HSV-1 gD. MW indicates the molecular weight marker, water indicates a no template control, and – indicates an empty well. **(C)** Nested PCR in US6 was performed on DNA extracted from EL75 and FB90 neural and lymphoid tissues (6 reactions per sample) alongside the equivalent of 100 or 10 genomes (viral particles, vp) of Macintyre (100 vp, 10 vp) quantitated viral load control. Tissues were trigeminal ganglia (TG), and palatine tonsil (PT). **(D)** Quality of the swab and tissue DNA was confirmed by GAPDH PCR (not nested, 230 base pair product) performed on the DNA sample.

Supplementary Figure 3 Productive oral HSV-1 infection in rhesus macaques is associated with recurrent shedding. **(A)** Results of nested PCR for HSV-1 US6 performed on each unclarified oral swab sample and the equivalent of 10 vp of Macintyre quantitated viral load control as in Supplementary Figure 2. These raw data correlate with the results presented in the heat map shown in Figure 1B. **(B)** Results of non-nested PCR for GAPDH performed for each of the unclarified oral swab samples. In some cases nested PCR results from HSV-1 US6 PCR of the Macintyre quantitated viral load control were run on the same gels (10 vp). **(C)** HSV-1 US6 nested PCR results from the trigeminal ganglia (TG) cultures. **(D)** GAPDH non-nested PCR results from the TG cultures.

Supplementary Figure 4 The limit of detection is lower for nPCR than qPCR**. (A)** Ten-fold dilutions of the Macintyre quantitated viral load control were subjected to the US6 nPCR to show the reduction in number of replicate reactions with amplification as a function of the amount of template. With only one genome per reaction, amplification still occurred in four of six reactions. **(B)** Ten-fold dilutions of the same Macintyre control DNA were subjected to the UL27 qPCR. The cut off for amplicon detection, C_T_ ≥ 35, was achieved between 30 and three genomes per reaction. **(C)** Amplicons from nested PCR were confirmed to be HSV-1 by sequencing the nested PCR product using the forward nesting primer. As an example, the published sequence of HSV-1 gD was aligned with sequence derived from nested PCR result for IB13 oral swab from 24 h post-HSV-1 infection (IB13 24 h swab) and IB13 trigeminal ganglia culture (IB13 TG).

Supplementary Figure 5 Oral HSV-1 infection produces little systemic cytokine or chemokine response.The concentration of soluble mediators (mean of duplicates) in each animal's plasma is shown over time following HSV-1 infection. Only those analytes in which the concentrations fell above the lowest standard are shown and concentrations that dipped down below the lowest standard are plotted as the lowest standard value. Oral swab cytokine data are presented in Figure 3.

Supplementary Figure 6 Gating strategy for T cell phenotype analysis. The frequency of live (LIVE/DEAD Aqua negative) CD3^+^CD4^+^ cells in blood expressing different surface markers is shown on day 0 prior to HSV-1 infection and on day 2 vs. the control sample from day 2 which was stained only for CD3 and CD4 (Control). For each marker, CD95 is on the x-axis and the indicated marker is on the y-axis. Cells shown are from DF98.

Supplementary Figure 7 HSV-specific T cells in HSV-1/SIV co-infected rhesus macaques are poorly activated. Data from Figure 5 were analyzed for co-expression of cytokine and CD69. **(A)** PBMCs and **(B)** splenocytes from GJ18 that secreted IL-2 and expressed CD69 in response to UL39 peptide pool (vs. DMSO and PMA/ionomycin) are shown. This animal had very few CD4^+^ T cells in the blood sample in accordance with its low CD4 count and high SIV viral load (as in Table 1 and Figure 2). **(C)** Data from cytokine co-expression with CD69 are shown for all animals in the CD4^+^ (top two panels) and CD8^+^ (bottom two panels) T cell compartments within blood (PBMC) and spleen (SPLN).

Supplementary Figure 8 TNF-α secretion by HSV-1-specific T cells. As in Figure 5, PBMCs collected 56 days post-HSV-1 infection were stimulated with pools of HSV-derived peptides spanning UL19, UL39, and US6 or with an HSV-1 lysate. PMA/ionomycin (P/I) served as the positive control. DMSO and mock virus lysate served as negative controls for peptides and virus lysate, respectively. The CD4^+^ and CD8^+^ responding cells secreting TNF-α are shown. CD4 is on the x-axis and TNF-α is on the y-axis.
