# Supplementary material for: Experimental Oral Herpes Simplex Virus-1 (HSV-1) Co-infection in Simian Immunodeficiency Virus (SIV)-Infected Rhesus Macaques
Source: Front Microbiol. 2017 Dec 5;8:2342. doi: 10.3389/fmicb.2017.02342 (PMC5723348; doi:10.3389/fmicb.2017.02342)
Supplement: Supplementary Figure 1 — Macaque oral tissues are susceptible to low-level HSV-1 F infection in vitro. Buccal and tonsil explants (3 × 3 × 3 mm) were exposed to HSV-1 F at 103, 104, 105, or 106 pfu/explant for 18 h. Explants exposed to 106 pfu/piece were cultured in the absence or presence of acyclovir (ACV). After extensive washing, tissues were cultured for 14 days. Supernatants collected over time were assayed for the presence of infectious virus by plaque assay on Vero cells. Tissues from three macaques were inoculated in triplicate for each tissue type and virus dose. The result of replicate cultures from each animal at each viral dose is shown (mean ± SEM). No virus grew in any culture containing ACV. Data from infection with 103 pfu were omitted from the graphs as no infection was observed. [file Presentation1.PDF]

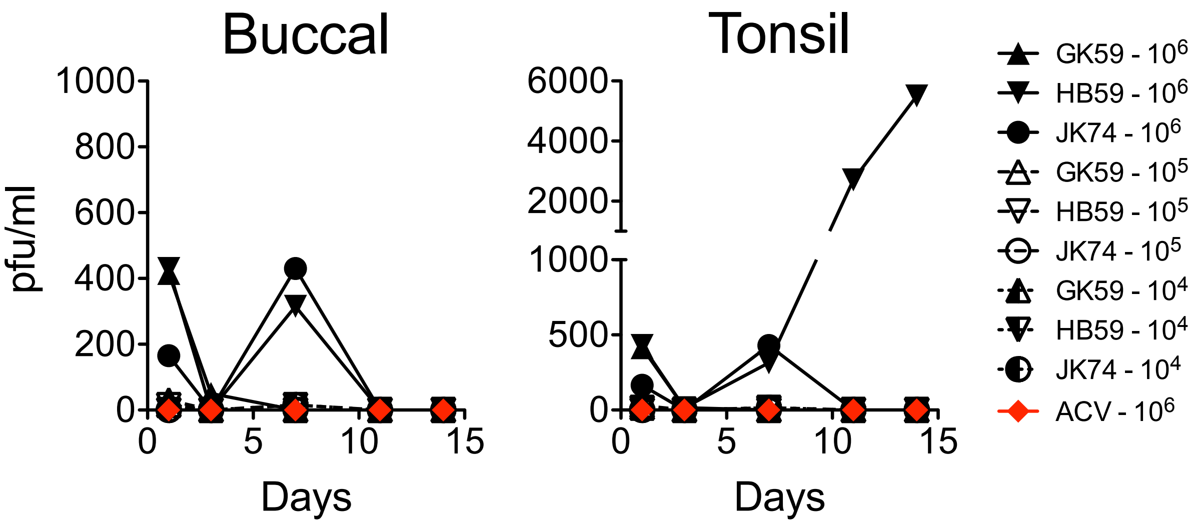

Supplementary Figure 1

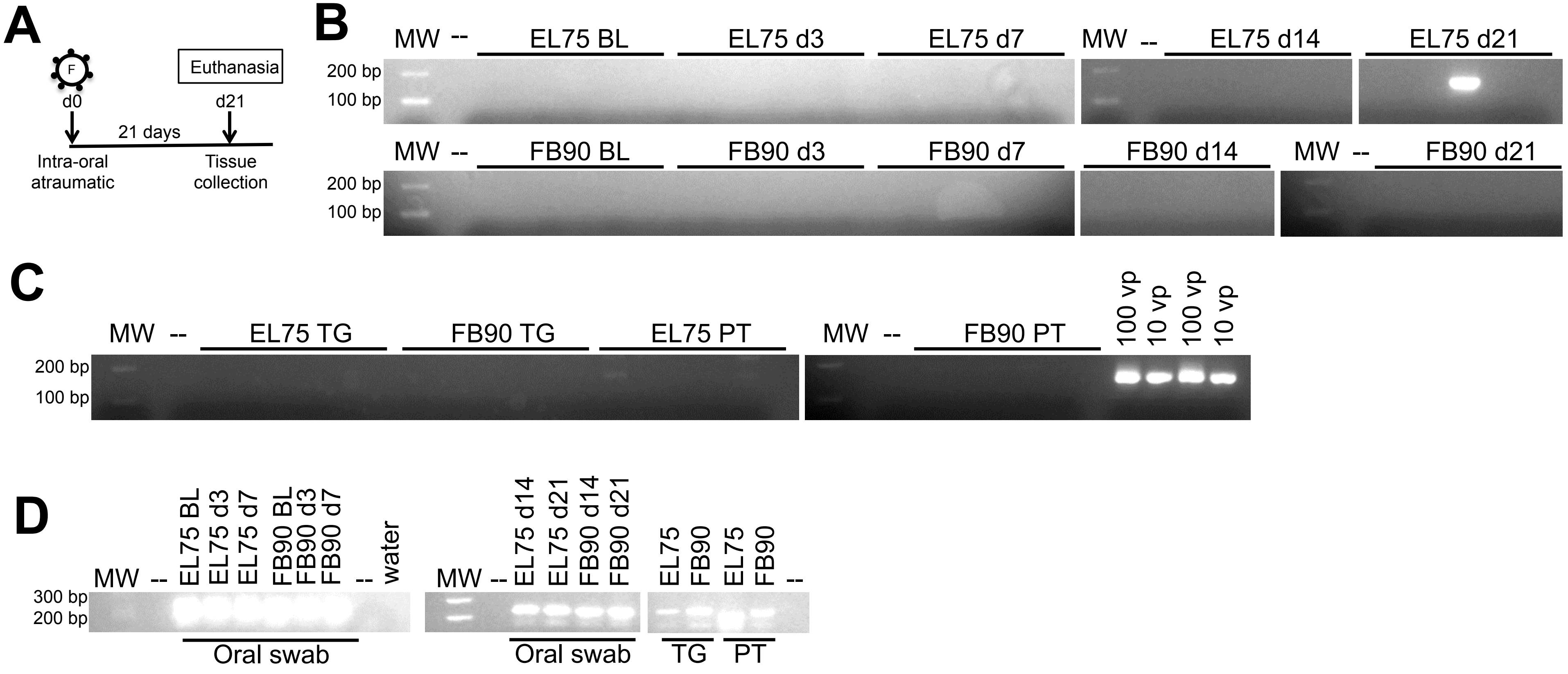

Supplementary Figure 2

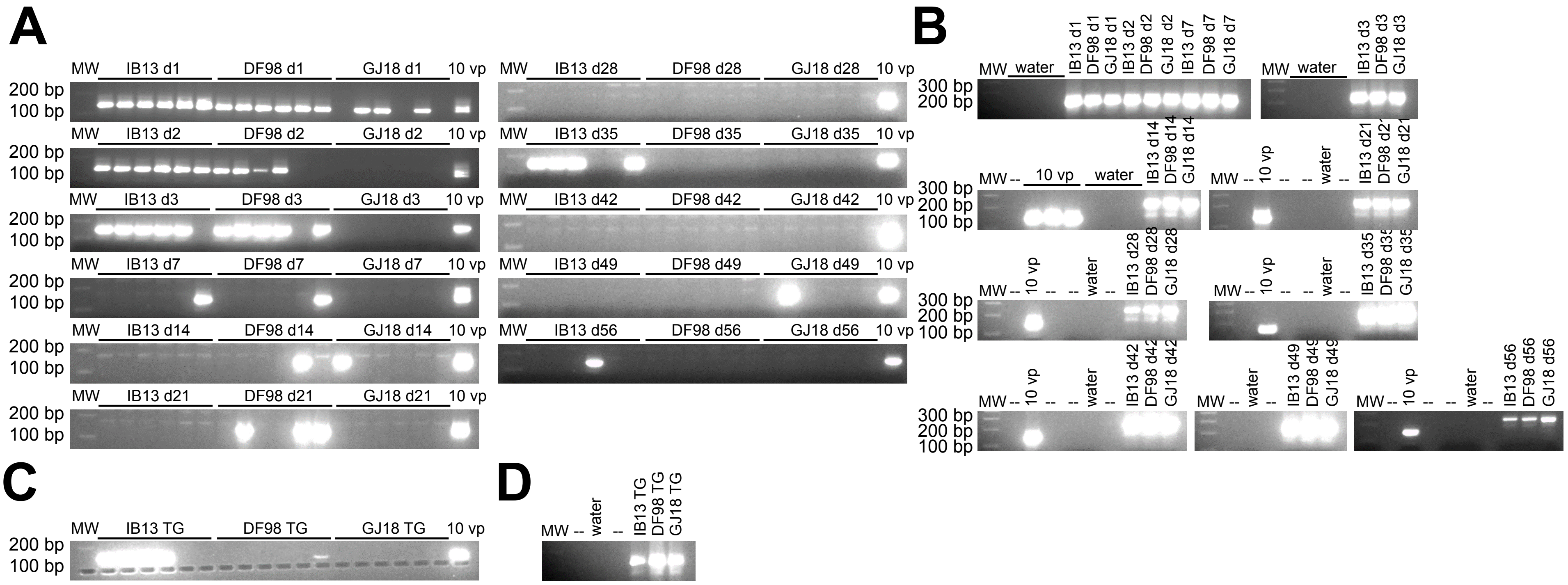

Supplementary Figure 3

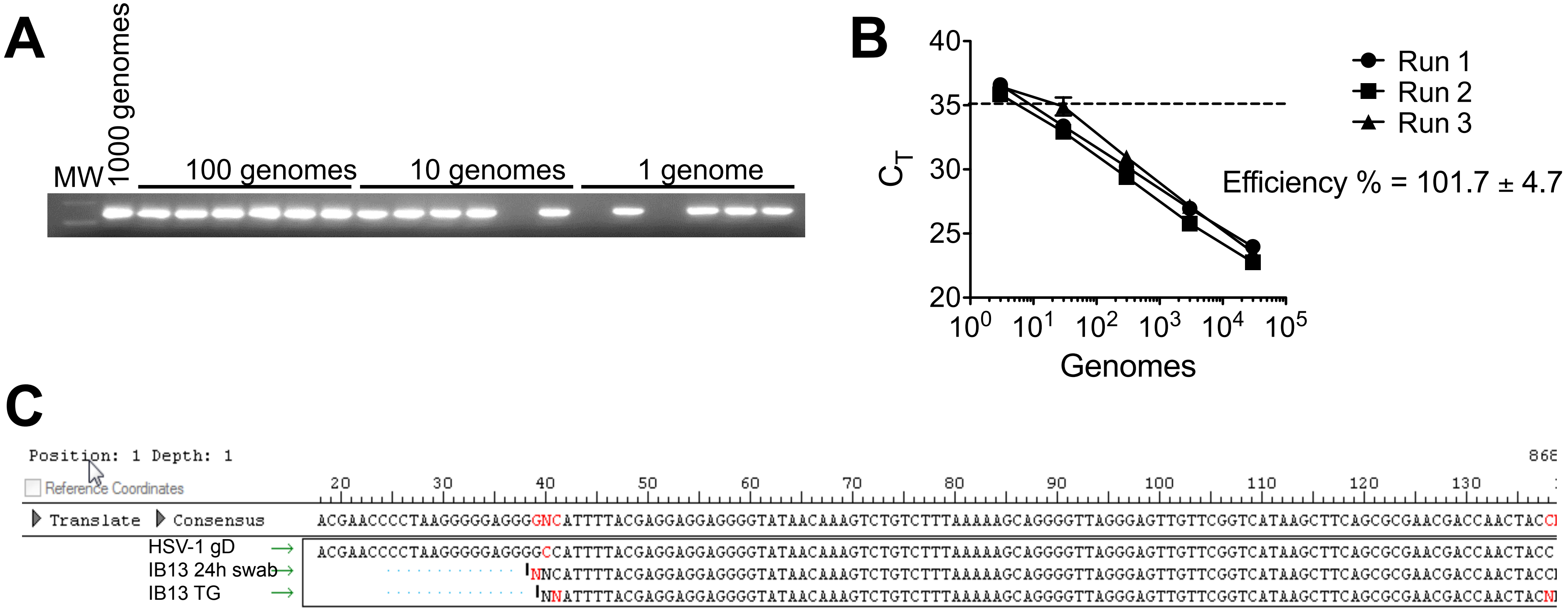

Supplementary Figure 4

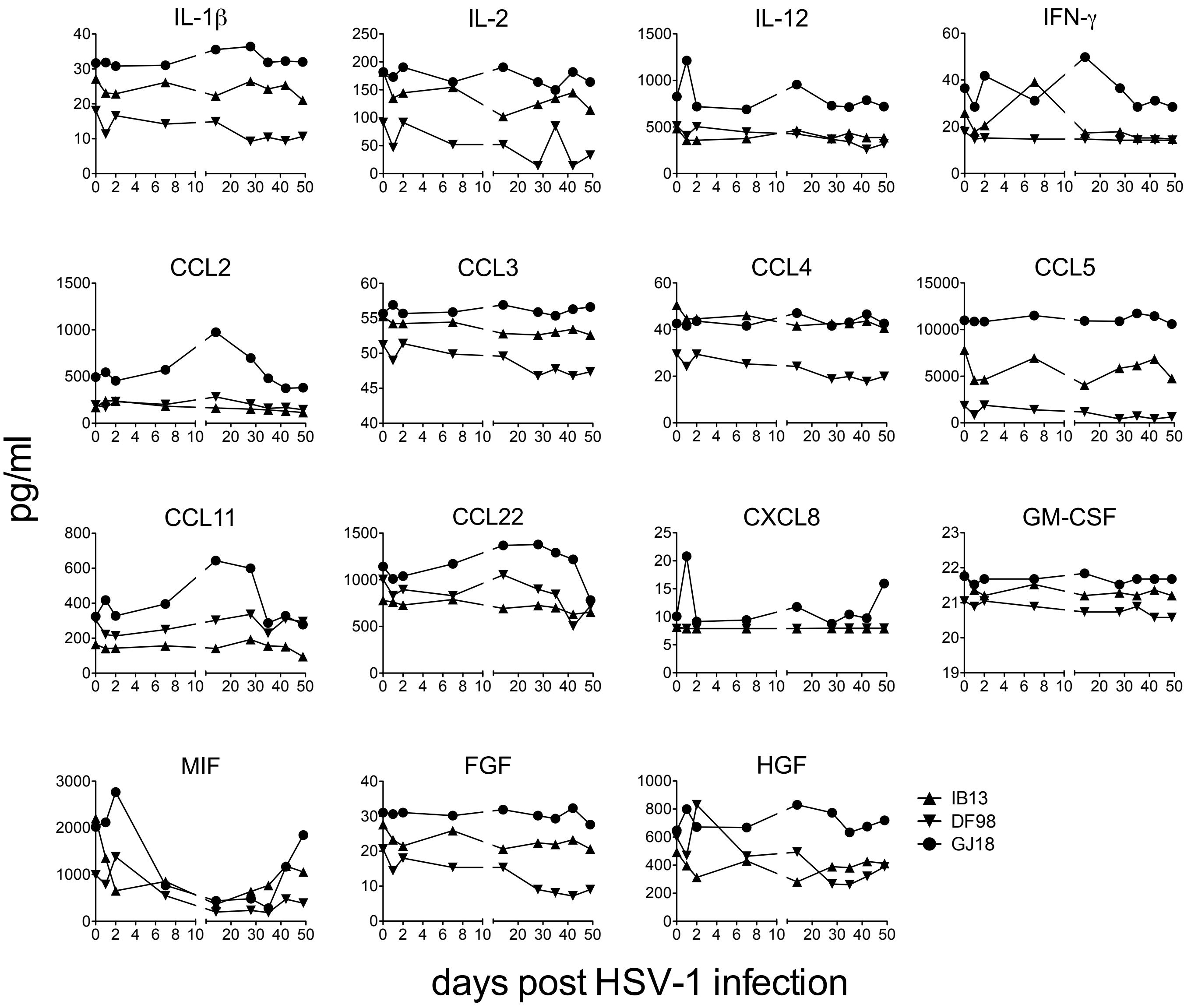

Supplementary Figure 5

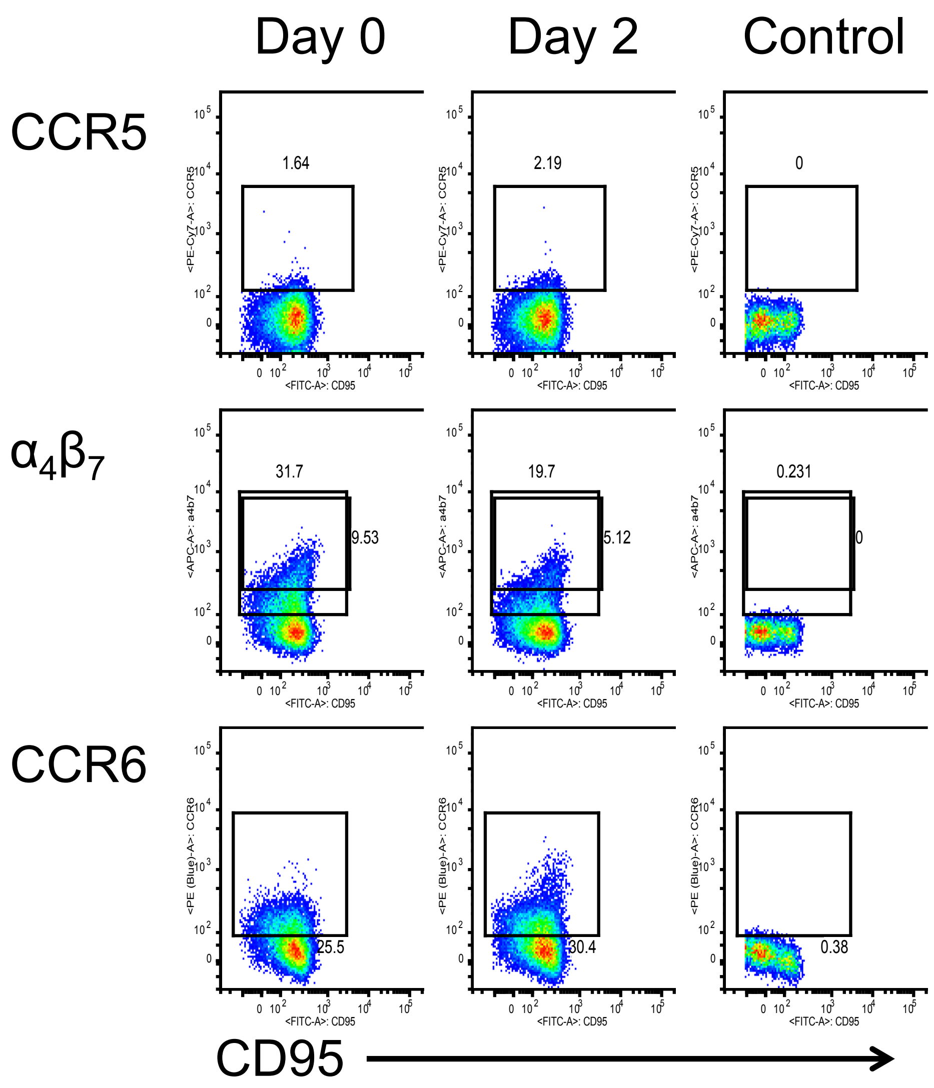

Supplementary Figure 6

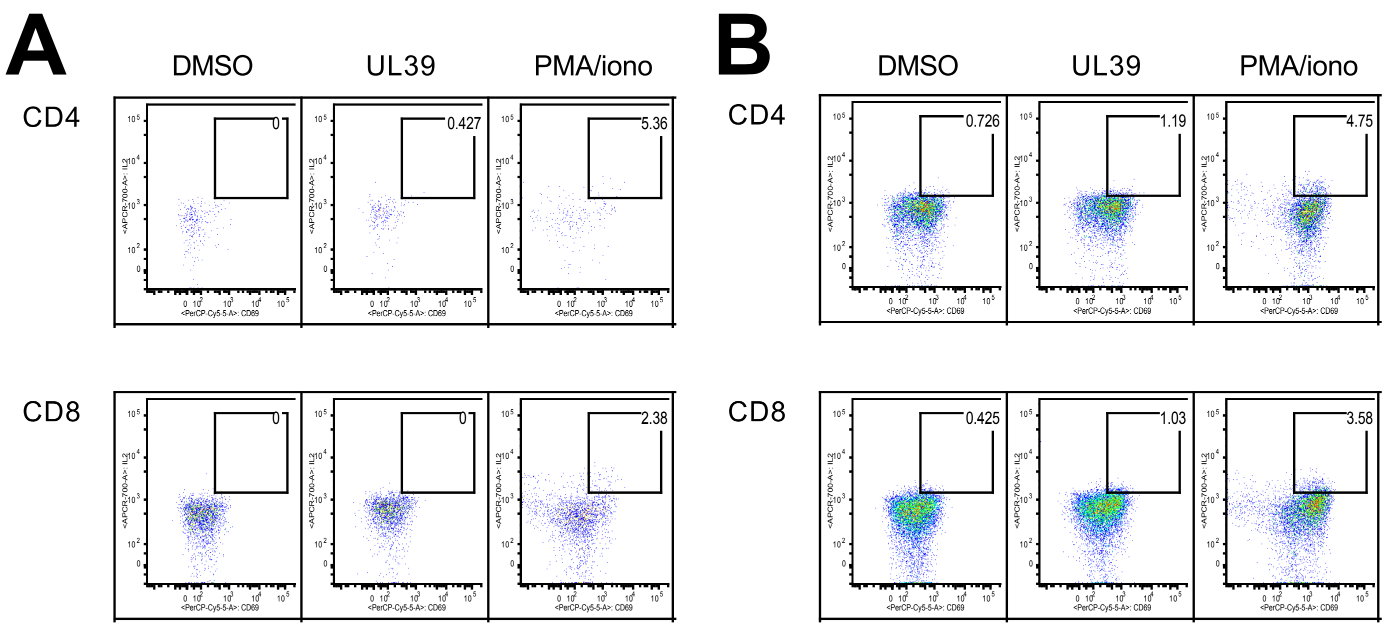

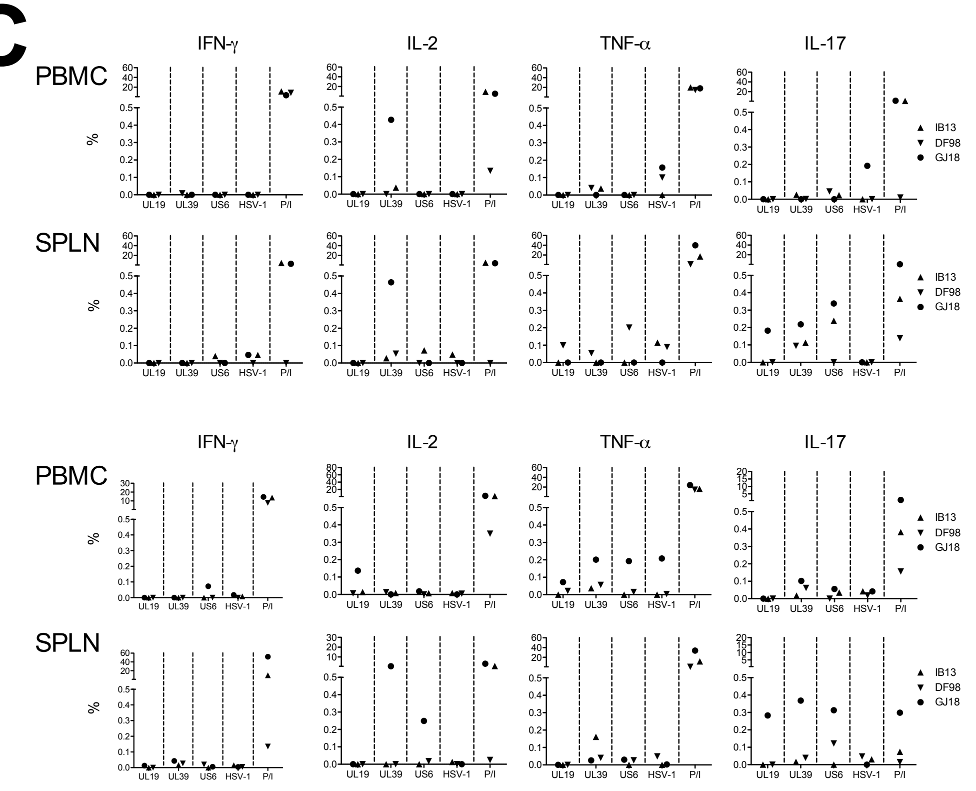

Supplementary Figure 7

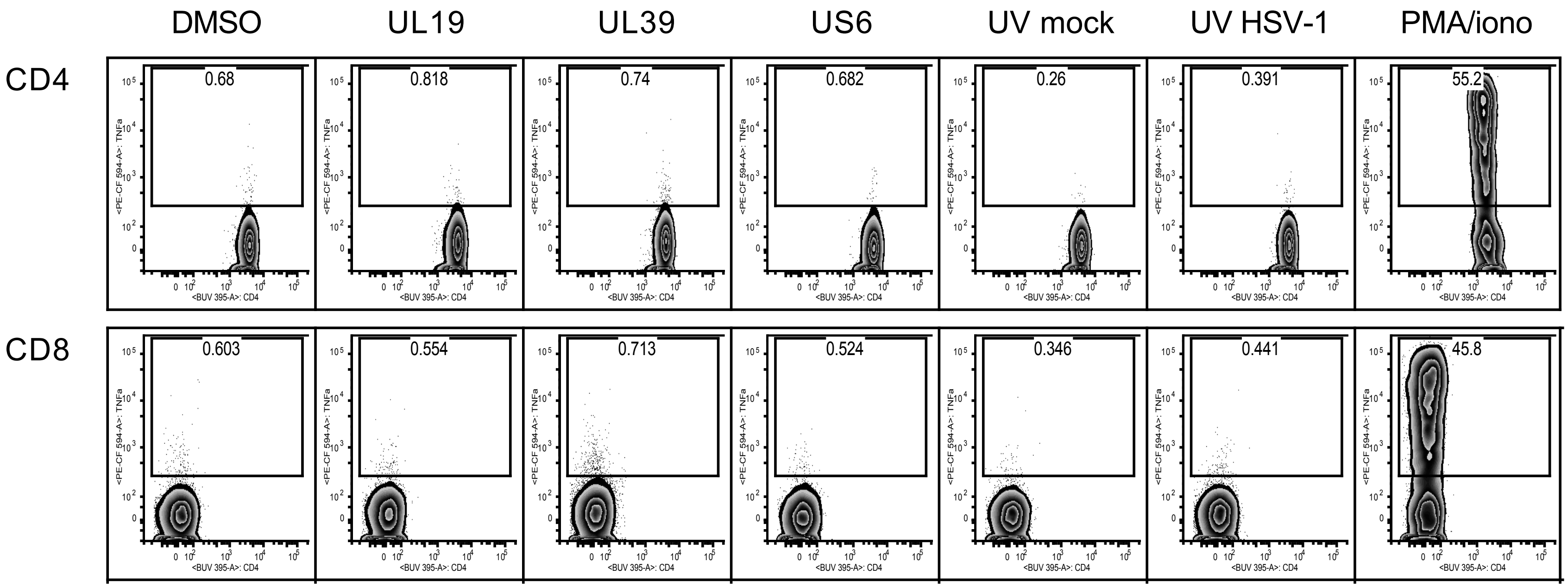

Supplementary Figure 8
